# Xanthones from the Bark of *Garcinia xanthochymus* and the Mechanism of Induced Apoptosis in Human Hepatocellular Carcinoma HepG2 Cells via the Mitochondrial Pathway

**DOI:** 10.3390/ijms20194803

**Published:** 2019-09-27

**Authors:** Shan Jin, Kuan Shi, Liu Liu, Yu Chen, Guangzhong Yang

**Affiliations:** 1 School of Pharmaceutical Sciences, South-Central University for Nationalities, Wuhan 430074, China; 2 College of Chemistry and Material Sciences, South-Central University for Nationalities, Wuhan 430074, China

**Keywords:** xanthones, *Garcinia xanthochymus*, apoptosis, HepG2, caspase, Bcl-2 family, MMPs

## Abstract

Xanthones are important chemical constituents of *Garcinia xanthochymus* and varied bioactivities including cytotoxicity. However, their anti-tumor mechanism has remained unknown. Here, we isolated and identified a new xanthone named garciniaxanthone I (**1**) and five known compounds from the bark of *G. xanthochymus*. Their structures were elucidated by NMR analysis and HRESIMS. The anti-proliferation activities of all isolated compounds were evaluated on four human tumor cell lines (HepG2, A549, SGC7901, MCF-7). The results demonstrated that the anti-proliferation activity of xanthone was related to the number and location of prenyl groups. We further found that garciniaxanthone I (GXI) could induce HepG2 apoptosis and enhance the expression of cleaved caspase-8, caspase-9, and caspase-3. GXI could also increase Bax level and concurrently reduce the overexpression of Bcl-2, Bcl-XL, Mcl-1, and surviving in HepG2 cells. Moreover, GXI could inhibit cell migration of HepG2 cells by inhibiting the expressions of MMP-7 and MMP-9. In summary, our study suggests that GXI could induce HepG2 apoptosis via the mitochondrial pathway and might become a lead compound for liver cancer treatment.

## 1. Introduction

There were 9.6 million cancer deaths in 2018, and liver cancer is one of the top five most common cancers [1]. Molecularly targeted therapy plays an important role in this treatment, and one of the most viable approaches is apoptosis [2]. Apoptosis is a type of programmed cell death. Nature uses it to stabilize the internal environment. However, apoptosis is often inhibited in cancer cells, and apoptosis induction in tumor cells could be an anti-tumor target [3]. Usually, apoptosis relies on a cascade reaction of the caspase family. When signal factors combine with transmembrane death receptors such as CD95, caspase-8/10 levels increase and act as a promoter. In some cases, signals transmit to effector caspase-3/7 via Bcl-2 families and cleaved caspase-9 through the mitochondria. This pathway also finally leads to cell death and is termed the mitochondria pathway [4,5]. Members of the Bcl-2 family are closely related to tumorigenesis, tumor progression, tumor metastasis, drug resistance, and prognosis. Therefore, Bcl-2 inhibitors will become a vital target of oncotherapy [6,7].

The genus *Garcinia* Linn. (Guttiferae) comprises about 450 species mainly distributed in Southeast Asia, South Africa, and West Polynesia. There are 21 species of Guttiferae that grow in Southeast China [8]. Xanthones are a characteristic component of Guttiferae and have anti-tumor, antibiosis, anti-malarial, antioxidant, anti-inflammatory, and antiviral activities [9]. Modern pharmacological research suggests that xanthones from Guttiferae have significant anti-tumor activities including caspase activation, apoptosis, and cell cycle arrest [10,11,12]. 

As a traditional folk medicine, *Garcinia xanthochymus* is used to clear “heat” and repel insects [8]. Recently, our team isolated 20 xanthones from *G. xanthochymus* and reported the anti-diabetic activity in L6 myotubes. The anti-diabetic activity was mainly dependent on promotion of glucose uptake and activation of PI3K/Akt and AMPK pathways [13]. Many studies have shown that xanthones from the bark of *G. xanthochymus* have cytotoxic activity, and the mechanism is unknown [3]. Thus, we decided to study the anti-tumor activity of all isolated xanthones. Here, we identified a new polyprenyl xanthone (**1**) and five known xanthones (**2**–**6**) from the bark of *G. xanthochymus*. We reported the anti-proliferation activities of all the 26 xanthones including 20 xanthones from this plant in our previous report [13]. That work used HepG2, A549, SGC7901, and MCF-7 cells. Here, we evaluate the anti-tumor mechanism of the new polyprenyl xanthone in HepG2 cells.

## 2. Results

### 2.1. Structural Elucidation of Isolated Compounds

**Figure 1:**
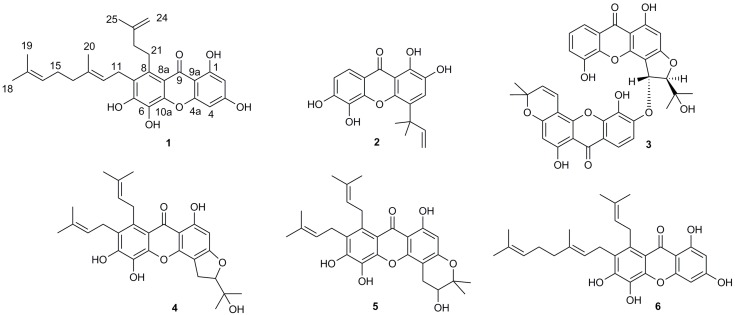
Structures of compounds **1**–**6.**

By means of standard isolation methods such as solvent extraction, silica column chromatography, and HPLC, a new xanthone named garciniaxanthone I (GXI) (**1**), as well as five known xanthones (**2**–**6**) (Figure 1), were isolated from the EtOAc extract of *G. xanthochymus* bark.

Compound **1** was isolated as a yellow oily matter and gave the molecular formula C_28_H_32_O_6_ by its HR-ESI-MS ([M + H]^+^ *m/z* 465.2273, calcd. 465.2277) and there were 13 degrees of unsaturation. As shown in Table 1, the ^13^C-NMR spectrum indicated the presence of four methyls, six methylenes, four methylenes, and fourteen quaternary carbons including a carbonyl signal [*δ_C_* 183.4]. The ^1^H-NMR spectrum revealed the presence of two isolated aromatic protons signals [*δ_H_* 6.19 (1H, s), 6.36 (1H, s)], a 3-methyl-3-butenyl group signal [*δ_H_* 3.44 (2H, m), 2.27 (2H, t, *J* = 8.0 Hz), 4.77 (1H, s), 4.84 (1H, s), 1.75 (3H, s)], a geranyl signal [ *δ_H_* 3.54 (2H, d, *J* = 6.0 Hz), 5.14(1H, t, *J* = 6.0 Hz), 2.00 (2H, m), 2.09 (2H, m), 5.07(1H, t, *J* = 7.0 Hz), 1.60 (3H, s), 1.56 (3H, s), 1.81 (3H, s)], and a chelated hydroxy signal [δ_H_ 13.68 (1H, s)]. Thus, we speculated that **1** was a xanthone bearing a 3-methyl-3-butenyl, a geranyl, and four hydroxy groups. We compared the NMR data of **1** with those of 7-geranyl-1,3,5,6-tetrahydroxy-8-(3-methyl-2-butenyl) xanthone (**6**) [14]. The results indicated that their structures were similar to each other except for the presence of 3-methyl-3-butenyl in **1** instead of 3-methyl-2-butenyl in **6**. Furthermore, the HMBC spectrum showed that the CH_2_-11 [*δ_H_* 3.54 (2H, d, *J* = 6.0 Hz)] of geranyl was correlated with C-6 (*δ_C_* 150.2), C-7 (*δ_C_* 125.8) and C-8 (*δ_C_* 135.8) and CH_2_-22 of 3-methylbut-3-enyl was correlated with C-8 (*δ_C_* 135.8). These data suggest that the 3-methyl-3-butenyl and the geranyl were located at C-8 and C-7, respectively. The HMBC correlations of H-2 [*δ_H_* 6.19 (1H, s)] with C-1 (*δ_C_* 165.1), C-3 (*δ_C_* 165.3), C-4 (*δ_C_* 93.8), and C-9a (*δ_C_* 103.9) as well as H-4 [*δ_H_* 6.36 (1H, s)] with C-2 (*δ_C_* 98.8), C-3 (*δ_C_* 165.3), C-4a (*δ_C_* 157.6), and C-9a (δ_C_ 103.9) confirmed the proposed structure. Thus, compound **1** was elucidated as 7-geranyl-1,3,5,6-tetrahydroxy-8-(3-methyl-3-butenyl) xanthone and named garciniaxanthone I (GXI). The ^1^H-NMR, ^13^C-NMR, HSQC and HMBC spectrum map of compound **1** was shown in Supplementary Materials Figures S1–S4.

The five known xanthones isolated from *G. xanthochymus* bark in this study were identified as symphoxanthone (**2**) [15], jacarelhyperols B (**3**) [16,17,18], garcinenones Y (**4**) [19], 3,4-dihydro-3,6,7,11-tetrahydroxy-8,9-di-(3-methyl-2-butenyl)-2,2-dimenthyl-pyrano-[2,3-c]xanthone (**5**) [20], and 7-geranyl-1,3,5,6-tetrahydroxy-8-(3-menthyl-2-butenyl)xanthone (**6**) [14] through comparison of the spectroscopic data with the literature. The NMR spectrum map of the known compounds was shown in Supplementary Materials Figures S5–S30.

### 2.2. Anti-Proliferation Activity on Human Tumor Cell Lines

The anti-proliferation activity of the 26 isolated xanthones was evaluated using the MTT assay on the human hepatocellular carcinoma cell line HepG2, the human lung adenocarcinoma cell line A549, the human gastric adenocarcinoma cell line SGC7901, and the human breast carcinoma cell line MCF-7, doxorubicin served as a positive control (Table 2). Compounds **7**–**26** were identified as 1,4,5,6-tetrahydroxy-7,8-di(3-methylbut-2-enyl) xanthone (**7**), 1,3,5,6-tetrahydroxy-4,7,8-tri(3-methyl-2-butenyl) xanthone (**8**), 1,5,6-trihydroxy-7,8-di(3-methyl-2-butenyl)-6′,6′-dimethylpyrano (2′,3′:3,4) xanthone (**9**), garcinenone D (**10**) 1,2,5-trihydroxylxanthone (**11**), 2,5-dihydroxy-1-methoxy xanthone (**12**), 1,5-dihydroxy-3-methoxy xanthone (**13**), 1,6-dihydroxy-4,5-dimethoxy xanthone (**14**), 12b-hydroxy-des-D-garcigerrin (**15**), 1,3,5,6-tetrahydroxy-8-(3-methylbut-2-enyl) xanthone (**16**), 1,4,6-trihydroxy-5-methoxy-7-(3-methylbut-2-enyl) xanthone (**17**), 1,3,5-trihydroxy-4-(3-methylbut-2-enyl)-9H-xanthen-9-one (**18**), garcinenone A (**19**), garcinexanthone B (**20**), 6-deoxyjacareubin (**21**), atroviridin(**22**), 1,2,5,6-tretrahydroxy-4-(1,1-dimethyl-2-propenyl)-7-(3-methyl-2-butenyl) xanthone (**23**), 1,3,5,6-tetrahydroxy-7-geranylxanthone (**24**), garcinenone E (**25**), and garciniadepsidone A (**26**) in our previous study [13], the structures of all compounds was shown in Supplementary Materials Figure S31.

The anti-proliferation result showed that compounds **1** and **3**–**10** had anti-proliferation activity on the four human tumor cell lines with IC_50_ values less than 50 μmol·L^−1^. Compound **8** showed the best anti-proliferation activity of all isolated xanthones. The preliminary SAR analysis demonstrated that xanthones with no prenyl group had low anti-proliferation activity. The activity strongly increases with two or more than two prenyl groups. These results suggest that polyprenylated xanthones might have an antitumor composition of *G. xanthochymus* and the activities might be related to the substitution of prenyl groups.

### 2.3. GXI Induced HepG2 Apoptosis

**Figure 2:**
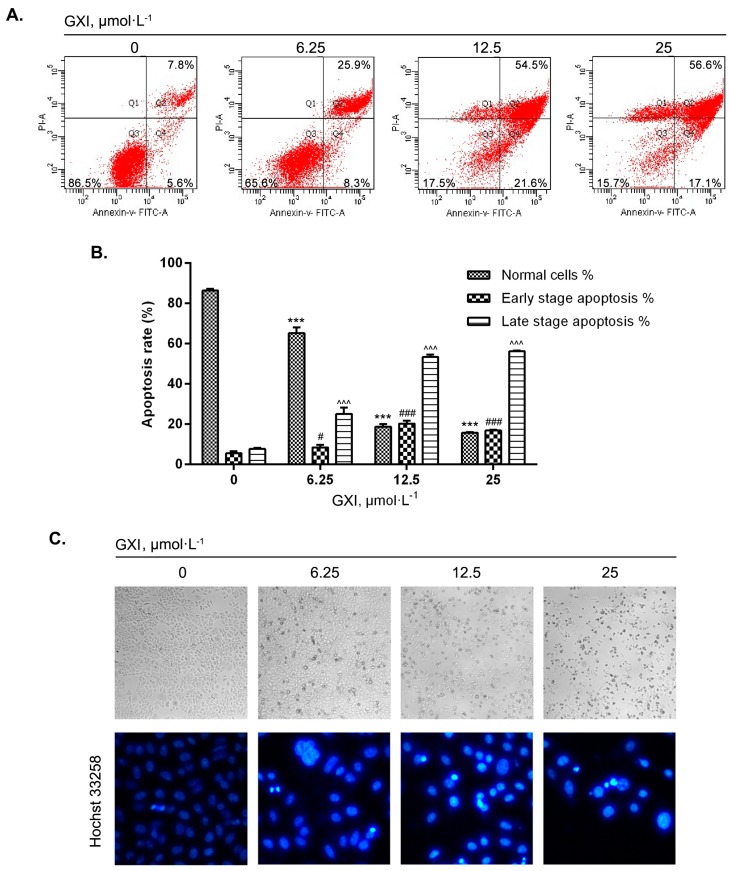
GXI-induced HepG2 apoptosis. (**A**) Apoptosis effects of dose-dependent treatment of GXI in HepG2 cells detected by Annexin V-FITC/PI staining. HepG2 cells treated with various doses of GXI for 12h. (**B**) Fold change of Annexin V-FITC/PI staining. Data represent mean ± SD. For normal cells, *** *p* < 0.001 compared with control, for early-stage apoptosis, # *p* < 0.05, ### *p* < 0.001 compared with control, for late-stage apoptosis, ^^^ *p* < 0.001 compared with control. (**C**) Morphological changes after treatment with various doses of GXI for 24 h analyzed by direct microscopy examination in 100× and Hochst 33258 fluorescent staining in 400×.

To investigate whether GXI in different doses could induce HepG2 apoptosis, Annexin V-FITC/PI staining was used to detect the apoptosis effect of HepG2 cells (Figure 2A). The cellular morphology changes due to apoptosis were observed with direct microscopy and Hochst 33258 fluorescent staining (Figure 2C). FCM analysis showed that the proportion of normal cells declined, and the proportion of apoptotic cells significantly increased with increasing GXI concentration. The early apoptosis rate at 25 μmol·L^−1^ was slightly lower than at 12.5 μmol·L^−1^. The late apoptosis and necroptosis rates increased so that the cells with a high dose of GXI might undergo late apoptosis faster or have more necroptosis (Figure 2B).

Direct microscopy showed that control cells grew well and arranged closely. As the GXI levels increased, there were more apoptotic cells that became round and floated in the medium. Cells in the control group had uniform fluorescent intensity. With increasing GXI levels, cells had nuclear chromatin condensation as seen via Hochst 33258 fluorescence. These observations confirmed that GXI could induce HepG2 apoptosis.

### 2.4. GXI-Induced HepG2 Apoptosis via the Mitochondrial Pathway

**Figure 3:**
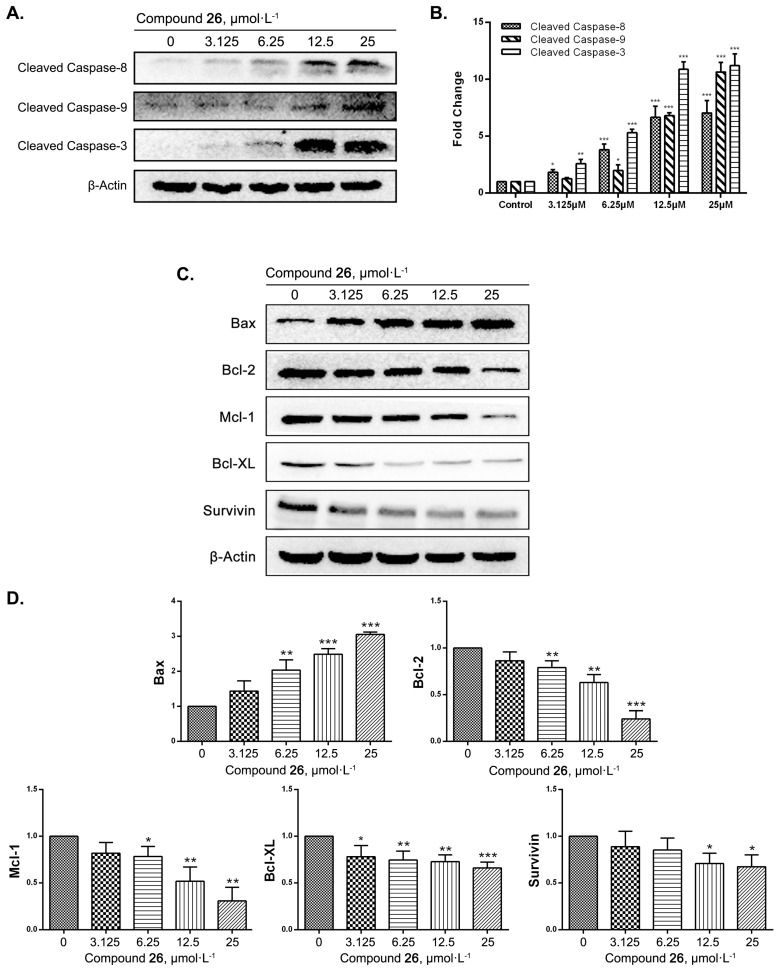
GXI induced HepG2 apoptosis via caspase cascade reaction and the mitochondrial pathway in HepG2 cells, HepG2 cells treated with various doses of GXI for 12 h. (**A**) Levels of cleaved caspase-8, cleaved caspase-9, and cleaved caspase-3 by Western blotting. (**B**) Fold change of Western blotting. Data represent mean ± SD. * *p* < 0.05, ** *p* < 0.01, *** *p* < 0.001 compared with control. (**C**) Levels of Bcl-2 family apoptosis-related proteins (Bax, Bcl-2, Mcl-1, Bcl-XL, Survivin and β-Actin) detected by Western blotting. (**D**) Fold change of Western blotting. Data represent mean ± SD. * *p* < 0.05, ** *p* < 0.01, *** *p* < 0.001 compared with control.

Western blots were done to determine whether GXI induced HepG2 apoptosis via mitochondrial pathways. We first detected the level of cleaved caspase-8, caspase-9, and caspase-3 after treatment with GXI at different concentrations for 12 h. The results showed that the level of cleaved caspase-8, caspase-9, and caspase-3 was notably lower after GXI treatment (Figure 3A,B). We then detected the level of Bax, Bcl-2, Mcl-1, Bcl-XL, and survivin in HepG2 after GXI treatment. As a result, the expression of the pro-apoptotic gene Bax dramatically increased, and the expression of apoptosis suppressor genes Bcl-2, Mcl-1, Bcl-XL, and survivin dramatically decreased after GXI treatment (Figure 3C,D).

### 2.5. GXI Induced HepG2 Migration

**Figure 4:**
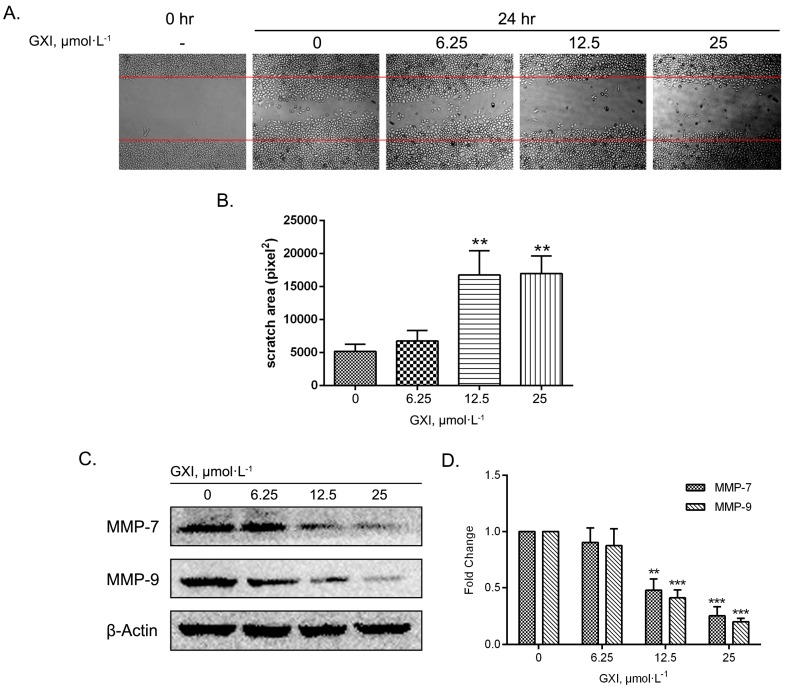
Inhibitory effects of GXI on cell migration in HepG2 cells. HepG2 cells treated with various doses of GXI for 24 h, and cell migration were tested by wound healing assay. (**A**) Cells migrating into the scratched area and photographed by inverted microscope in 100×. (**B**) Calculated as pixel^2^ of scratch area. Data represent mean ± SD. ** *p* < 0.01 compared with control. (**C**) Levels of MMP-7, MMP-9, and β-Actin detected by Western blotting. (**D**) Fold change of Western blotting. Data represent mean ± SD. ** *p* < 0.01, *** *p* < 0.001 compared with control.

A cell migration assay was performed via the wound healing method. The growth of cells beside the scratch was inhibited more with 24 h of GXI treatment than the control group. Statistical analysis showed that the scratch area with 12.5 and 25 μmol·L^−1^ GXI treatment had a significant difference versus the control group. Thus, these GXI concentrations could induce migration of HepG2 cells (Figure 4A,B). To study the anti-migration mechanism, MMP-7 and MMP-9 were detected via a Western blotting assay. The results showed that the expression of MMP-7 and MMP-9 significantly reduced after treatment with 12.5 and 25 μmol·L^−1^ GXI for 24 h (Figure 4C,D).

## 3. Discussion

In this study, we reported a new xanthone and five known xanthones from the bark of *G. xanthochymus*. This new polyprenyl xanthone was named garciniaxanthone I (**1**). Their structures were identified by extensive spectroscopic analysis. Prior work has shown that xanthones from *G. xanthochymus* have cytotoxic activity toward tumor cell lines [3,10,11,12]. Thus, we tested the anti-proliferation activities of all isolated xanthones on four typical types of human tumor cell lines (HepG2, A549, SGC7901, and MCF-7). The results showed that there were nine xanthones with broad-spectrum anti-tumor activities. Some of these xanthones had stronger anti-proliferation activities than doxorubicin. Han used SAR analysis to show that the extra prenyl groups led to a higher cytotoxic activity. Prenylation at the nucleus of the xanthone led to more activity than at the side chain [21]. Our study verified these ideas: Compound **8** has three prenyl groups on the nucleus and had four-fold more anti-proliferation activity than xanthones with a prenyl group on the side chain. Xanthones with no prenyl groups also had no anti-proliferation activity.

Since there were only cytotoxicity studies of xanthones from G. xanthochymus at present, we decided to study the anti-tumor mechanism of the new polyprenyl xanthone GXI. To study whether GXI could induce HepG2 apoptosis, we started our study from the phenomenon of apoptosis. The morphological changes of apoptosis had some regularity such as shrink of cell volume, nuclear pyknosis, metachromatic condensed, the disappearance of the mitochondrial membrane potential, and apoptosis bodies forming [22]. Thus, we used fluorescent staining to detect the effects of apoptosis. In this part of the study, we observed nuclear chromatin condensation of HepG2 cells by Hochst 33258 staining, and we detected the rising of apoptosis rate by FCM assay after treated with GXI. All these results proved that GXI could induce apoptosis of HepG2 cells.

We further studied the mechanism of GXI-induced HepG2 apoptosis, which relies on caspase induction. Inducing tumor cell apoptosis was an effective measure in tumor targeting therapy, as we knew that apoptosis relied on caspase cascade reaction. Meanwhile, the Bcl-2 family members played important roles in this process. In the mitochondrial pathway of apoptosis, BH-3 interacts with the death agonist (Bid) and is cleaved to tBid via activated caspase-8. The tBid is then transported to the mitochondria where it regulates Bax. Cytochrome C is then released into the cytoplasm and combines with the caspase-9 activator Apaf-1 so that caspase-9 would be activated to finally activate caspase effectors like caspase-3 leading to apoptosis [23,24]. 

**Figure 5:**
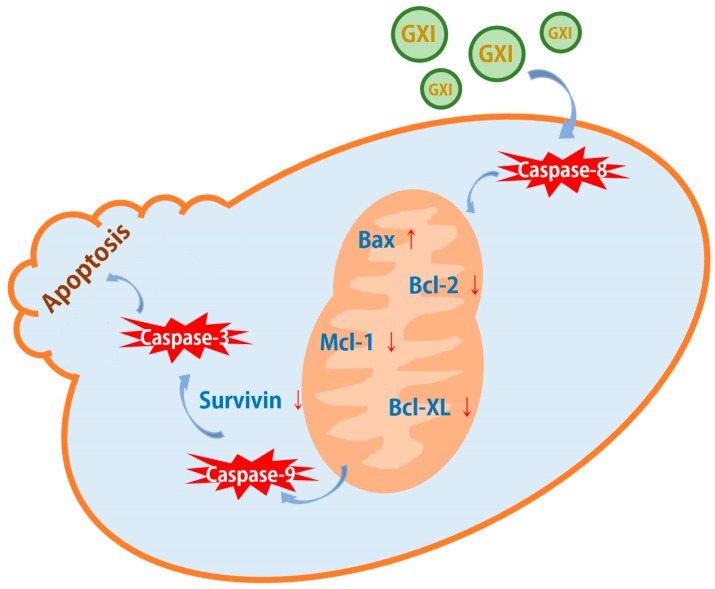
GXI-induced apoptosis via the mitochondrial pathway.

Our data show that the expression of the apoptosis promoter cleaved caspase-8 and effector cleaved caspase-3 was significantly elevated after treatment with GXI. As a pivotal kinase of the mitochondrial pathway, caspase-9 was significantly activated after also being treated with GXI. Then the level of Bcl-2 family pro-apoptotic protein Bax could significantly be up-regulated after GXI treatment had been detected. Finally, we detected the level of Bcl-2 family anti-apoptotic protein Bcl-2, Bcl-XL, Mcl-1, and Survivin, these proteins were overexpressed in tumor cells and helped tumor cells avoid apoptosis [25,26]. GXI could downregulate the expressions of these proteins and had concentration dependence. These studies suggest that GXI could induce HepG2 apoptosis via the mitochondrial pathway. After GXI treatment, caspase-8 would be activated first. Its signal was then conducted into the mitochondria to regulate the Bcl-2 family. The signal from the mitochondria was then transferred to the cytoplasm to activate caspase-9 and, in turn, activate the apoptosis effector caspase-3 (Figure 5).

Many xanthones had anti-tumor activity on several human tumor cell lines. Liu studied the apoptosis mechanism of two xanthones, their results showed that these xanthones could activate caspase-9 and caspase-3 [27]. Moreover, Wu’s study showed that a new xanthone could induce HepG2 apoptosis via the mitochondrial pathway, the apoptosis relied on caspase-9/7/3 activation, Bcl-2 downregulation and Bax up-regulation [12]. All these conclusions were similar to our study.

Furthermore, we also studied GXI’s inhibition of cell migration because migration underlies metastasis. Matrix metalloproteinases (MMPs) are elevated in some tumor cells. The mechanism of metastasis is complicated, but some studies have shown that MMPs could degrade the extracellular matrix (ECM) leading to the secretion of EGF, TGF-β, and IGF and thus metastasis [28,29]. MMP-7 is a matrix-degrading enzyme. MMP-9 is also known as gelatinase B.

Roeb’s study proved that the enhanced expression of MMP-7 and gelatinase was a feature of tumorigenesis [30]. Herszenyi verified that invasion depth and metastasis distance had positive correlations with MMP-9 [31]. Since α-mangostin is a typical polyprenyl xanthone in Guttiferae, many studies chose α-mangostin to study the bioactivities of xanthone, previous studies showed that α-mangostin could inhibit cell migration in various human cancer cells, like human prostate carcinoma PC-3, human breast carcinoma MCF-7, and human lung adenocarcinoma A549 cells, and the mechanism involved the inhibition of MMPs [32,33,34]. Like α-mangostin, we found that GXI could inhibit cell migration as well as MMP-7 and MMP-9 expression in HepG2 cells. Thus, GXI might inhibit metastasis of liver cancer via MMP-7 and MMP-9 inhibition. These studies suggest that polyprenyl xanthone might be a good lead compound for anti-cancer drugs.

## 4. Materials and Methods

### 4.1. General

The 1D and 2D NMR spectra were recorded on a Bruker AVANCE Ⅲ-500 MHz and a Bruker AV-400 MHz spectrometer (Bruker, Ettlingen, Germany) in pyridine-d_5_ or acetone-d_6_ using tetramethyl silane (TMS) as an internal reference standard. Chemical shifts (δ) are expressed in ppm, and the coupling constants (*J*) are given in Hz. High-resolution electrospray mass spectroscopy was performed on a Waters Autospec Premier 776 mass spectrometer (HR-EI-MS) (Waters Technologies, Milford, MA, USA) and an Agilent G6230 TOF mass spectrometer (HR-ESI-MS) (Agilent Technologies, Santa Clara, CA, USA). High-performance liquid chromatography (HPLC) was conducted on an Ultimate 3000 HPLC system (Thermo Fisher, Waltham, MA, USA) equipped with an Ultimate 3000 pump and Ultimate 3000 variable wavelength detector, as well as a semi-preparative YMC-Pack ODS-A column (250 × 10 mm, 5 μm) from YMC Co. Ltd. (Kyoto, Japan), column chromatography (CC) was conducted over silica gel (200–300 mesh and 300–400 mesh, Qingdao Haiyang Chemical Industry, Qingdao, China). Chromatographic grade methanol and acetonitrile were purchased from Tedia Co. Inc. (Fairfield, OH, USA). 

### 4.2. Plant Material

All plant materials were collected from Xishuangbanna Dai Autonomous Prefecture, Yunnan province, P. R. China, and identified as dried barks of *Garcinia xanthochymus* by chief pharmacist Yinghong Zhao from Xishuangbanna Prefecture National Medicine Research Institute. The voucher specimen (No. 20120915) was deposited in the herbarium of the School of Pharmaceutical Sciences, South Central University for Nationalities, P.R. China.

### 4.3. Extraction and Isolation

The powered bark of *G. xanthochymus* (7.9 kg, dry wt.) was extracted thrice with 95% EtOH at room temperature (each time for 24 h) to obtain 1.5 kg of EtOH extract, and then successively partitioned with petroleum ether (P. E.), EtOAc, and n-BuOH to obtain a P. E. extract (93.1 g), EtOAc extract (473 g), and n-BuOH extract (151 g). The EtOAc extract (330 g) was chromatographed over silica gel P.E./acetone (9:1, 8:2, 7:3, 5:5, 3:7, 0:1, v/v) to yield 12 fractions (Fr.1-Fr.12). Fr.6 (10.34 g) was subjected to a CC with cyclohexane/acetone (50:1→3:7, v/v) and then purified by ODS with H_2_O/MeOH (8:2→2:8, v/v), semi-preparative HPLC gave compounds **5** (6.6 mg) and **6** (50.0 mg). Fr.7 (8.62 g) was subjected to a CC with CH_2_Cl_2_/MeOH (1:1→4:6, v/v), and then purified with ODS and H_2_O/MeOH (7:3→2:8, v/v), and semi-preparative HPLC gave compounds **1** (3.3 mg), **2** (3.0 mg), **3** (1.8 mg), and **4** (4.3 mg).

Characterization: Garciniaxanthone I (**1**), yellow oil. UV (MeOH) λ max nm (logε): 203 (4.39), 245 (sh, 4.06), 299 (3.97), and 322 (sh, 3.89), ^1^H- and ^13^C-NMR: See Table 1. HR-ESI-MS *m*/*z*: 465.2273 [M + H]^+^ (calcd. for C_28_H_33_O_6_^+^: 465.2277).

### 4.4. Cell Culture

The human hepatocellular carcinoma cell line HepG2 cells, human lung adenocarcinoma cell line A549 cells, human gastric adenocarcinoma cell line SGC7901 cells, and human breast carcinoma cell line MCF-7 cells were bought from the cell bank of Chinese Academy of Sciences and cultured in Dulbecco’s modified Eagle medium (DMEM) (Hyclone, South Logan, UT, USA), supplemented with 10% fetal bovine serum (FBS) (Gibco, Grand Island, NY, USA) and 1% penicillin-streptomycin solution (Hyclone, South Logan, UT, USA). All these cell lines were obtained within six months before the experiments were performed and passed by STR analysis.

### 4.5. Cytotoxicity Assay

The cytotoxic effects of the compounds were evaluated by an MTT assay. Cells (HepG2, SGC7901, A549, MCF-7) were seeded in 96-well plates and incubated for 24 h and treated with different concentrations of compounds (50, 25, 12.5, 6.25, and 3.125 μmol·L^−1^) at 37 °C in 5% CO_2_ for 24 h. Due to the exorbitant activity, compound **8** was treated at lower concentrations (3.125, 1.563, 0.781, 0.391, and 0.195 μmol·L^−1^) in the second round of experiments. After treatment, 10 μL of MTT (Sigma-Aldrich, St. Louis, MO, USA) (5 mg·mL^−1^) was dissolved in DMEM for each well followed by incubation for 2–4 h. The medium was aspirated, and the formazan crystals were dissolved in 100 μL of DMSO (Sigma-Aldrich, St. Louis, MO, USA). The optical density (OD) of each well at 492 nm was determined with a Multiskan GO microplate reader (Thermo Fisher, Waltham, MA, USA) in 30 min. The cell viability in response to treatment was calculated as the percentage of control cells treated with solvent DMSO at the final concentration of 0.1%: Cell viability (%) = (100 × OD treated cells)/OD control cells. Doxorubicin hydrochloride (Aladdin, Shanghai, China) was used as positive control.

### 4.6. Fluorescence Staining Assay

The Hochst 33258 fluorescence staining assay determined the chromatin morphological changes of HepG2 Cells after GXI treatment. GXI was treated with various doses (6.25, 12.5, and 25 μmol·L^−1^) in HepG2 cells (1 × 10^6^ cells/mL) for 24 h. The medium was then removed, followed by 0.5 mL 75% ethanol for 10 min. After the fixation, ethanol was removed, and 0.5 mL Hochst 33258 (2 μg·mL^−1^) (Solarbio, Beijing, China) was added into the wells and treated for 5 min in a dark room. Pictures were taken by a fluorescent inverted microscope (Caikon, Shanghai, China). The excitation wavelength was 350 nm.

### 4.7. FACS Analysis

HepG2 cells were treated with different concentrations of GXI (6.25, 12.5, 25 μmol·L^−1^) for 12 h. Cell suspensions were prepared in EP tubes and washed three times with PBS and finally removed the PBS was by centrifugation. Next, 195 μL Annexin V-FITC binding buffer was added into the tubes, 5 μL Annexin V-FITC and 10 μL PI (BD, CA, USA) were added into the tubes after re-suspending the cells. The samples were incubated for 20 min in the dark. The Annexin V-FITC and PI stained cells were analyzed using Cellquest software with an LSR Ⅱ flow cytometer (BD, Franklin Lakes, NJ, USA).

### 4.8. Western Blotting

HepG2 cells were lysed in RIPA buffer (containing 1 mmol·L^−1^ PMSF) (Beyotime, Shanghai, China). The protein samples were quantified using an enhanced BCA assay kit (Beyotime, Shanghai, China). Proteins were separated on a 10% or 15% SDS-PAGE gel by electrophoresis and then transferred to a 0.45 or 0.22 μm PVDF membrane (Millipore, Darmstadt, Germany). The membrane was blocked with 5% skim milk and incubated with primary antibodies for caspase-8, caspase-9, caspase-3, Bax, Bcl-2, Mcl-1, Bcl-XL, survivin, MMP-7, MMP-9, and β-Actin (ABclonal, Wuhan, China) overnight at 4 °C and washed with TBST buffer three times. We then incubated the samples with HRP-conjugated goat-anti-rabbit IgG secondary antibody (ABclonal, Wuhan, China) and then washed with TBST buffer three times. Finally, we applied 1 mL of ECL agent (Advansta, Menlo Park, CA, USA) on the membrane, and the protein blots were developed using an Omega Lum G imaging system (Aplegen, Pleasanton, CA, USA). Image J software was used to scan the gray values of the blots.

### 4.9. Wound Healing Assay

The wound healing assay analyzed cell migration. HepG2 cells (1 × 10^6^ cells/mL) were seeded into a six-well plate and incubated to confluence at 37 °C. An area was then scratched with a sterile 200-μL pipette tip and washed with PBS. The cells were treated with different concentrations of GXI (6.25, 12.5, 25 μmol·L^−1^) in DMEM medium within 2% FBS for 24 h. After incubation, cells were photographed randomly under an inverted microscope, and the wound areas were analyzed by Image J software.

### 4.10. Statistical Analysis

Graphpad Prism 6 software was used for statistical analysis. All data were shown as mean ± SD. Statistically significant differences were evaluated with a two-way ANOVA analysis.

## 5. Conclusions

*G. xanthochymus* is not only an edible fruit but also a folk medicine in Southeast Asia. The anti-tumor activity of *G. xanthochymus* has been researched extensively in recent years. As a characteristic component of Guttiferae, xanthones have various bioactivities, but there is little anti-tumor research on xanthones in *G. xanthochymus*—especially mechanistic research. This is the first study to examine the anti-tumor mechanism in HepG2 cells via the xanthones from *G. xanthochymus*. In summary, we showed that nine xanthones from *G. xanthochymus* bark had broad-spectrum anti-tumor activities. We studied the SAR at the same time. We also demonstrated the anti-tumor mechanism by which GXI could induce HepG2 apoptosis via the mitochondrial pathway and had the ability to inhibit HepG2 cell migration by downregulating the expression of MMP-7 and MMP-9. However, this remains a preliminary study for anti-tumor activity and the mechanism of xanthone. Thus, further studies of xanthones on liver cancer treatment are necessary.

## Figures and Tables

**Table 1:** ^1^H NMR (500 MHz), ^13^C NMR (125 MHz) and HMBC data of compound **1** (Acetone-*d*_6_) ^1^.

Position	δ_H_	δ_C_	HMBC (H→C)
C-1		165.1	
C-2	6.19 (1H, s)	98.8	4, 9a, 1, 3
C-3		165.3	
C-4	6.36 (1H, s)	93.8	2, 9a, 4a, 3
C-4a		157.6	
C-10a		146.5	
C-5		130.8	
C-6		150.2	
C-7		125.8	
C-8		135.8	
C-8a		111.9	
C-9		183.4	
C-9a		103.9	
C-11	3.54 (2H, d, *J* = 6.0 Hz)	25.2	12, 13, 8, 6, 7
C-12	5.14 (1H, t, *J* = 6.0 Hz)	124.2	20, 11, 14
C-13		135.4	
C-14	2.00 (2H, m)	40.5	20, 15, 12, 13
C-15	2.09 (2H, m)	27.4	14, 16, 17
C-16	5.07 (1H, t, *J* = 7.0 Hz)	125.1	18, 19, 14
C-17		131.8	
C-18	1.60 (3H, s)	25.9	19, 16, 17
C-19	1.56 (3H, s)	17.8	18, 16, 17
C-20	1.81 (3H, s)	16.6	14, 12, 13
C-21	3.44 (2H, m)	29.2	
C-22	2.27 (2H, t, *J* = 8.0 Hz)	39.9	25, 21, 24, 8, 23
C-23		147.3	
C-24	4.84 (1H, s) 4.77 (1H, s)	110.2	25, 22, 23
C-25	1.75 (3H, s)	22.8	22, 24, 23
1-OH	13.68 (1H, s)		1, 2, 9a

^1^ The chemical shifts have been expressed in δ ppm. The coupling constants (*J*) have been expressed in Hz.

**Table 2:** Anti-proliferation activity of isolated xanthones ^1^.

Compounds ^2^	HepG2	A549	SGC7901	MCF-7
Doxorubicin ^3^	6.52 ± 0.13	14.03 ± 0.21	7.54 ± 1.11	4.40 ± 1.17
**1**	24.61 ± 1.89	50.67 ± 4.41	28.31 ± 3.10	17.81 ± 6.91
**3**	35.06 ± 2.10	38.14 ± 0.06	25.58 ± 6.99	38.50 ± 9.28
**4**	19.71 ± 6.03	22.27 ± 2.14	10.15 ± 1.30	10.67 ± 7.85
**5**	15.88 ± 6.45	24.99 ± 7.67	5.74 ± 3.02	7.09 ± 1.26
**6**	20.47 ± 2.00	5.29 ± 1.34	16.58 ± 2.64	5.25 ± 2.96
**7**	23.12 ± 1.85	20.46 ± 1.45	16.04 ± 0.29	47.50 ± 2.11
**8**	4.14 ± 0.39	10.54 ± 0.25	0.07 ± 0.01	2.37 ± 1.38
**9**	23.62 ± 2.60	5.61 ± 0.60	13.11 ± 1.88	13.90 ± 0.58
**10**	20.41 ± 0.92	9.28 ± 2.01	6.96 ± 0.73	9.31 ± 1.19

^1^ The results were shown as IC_50_ ± SD in μmol·L^−1^. ^2^ Other isolates with IC_50_ > 50 μmol·L^−1^ for all cell lines are not listed. ^3^ Doxorubicin was used as a positive control.

## Supplementary Materials

Supplementary materials can be found at https://www.mdpi.com/1422-0067/20/19/4803/s1. Figure S1. ^1^H-NMR spectrum of compound **1**. Figure S2. ^13^C-NMR spectrum of compound **1**. Figure S3. HSQC spectrum of compound **1**. Figure S4. HMBC spectrum of compound **1**. Figure S5. ^1^H-NMR spectrum of compound **2**. Figure S6. ^1^H-NMR spectrum of compound **3**. Figure S7. ^1^H-NMR spectrum of compound **4**. Figure S8. ^1^H-NMR spectrum of compound **5**. Figure S9. ^1^H-NMR spectrum of compound **6**. Figure S10. ^1^H-NMR spectrum of compound **7**. Figure S11. ^1^H-NMR spectrum of compound **8**. Figure S12. ^13^C-NMR spectrum of compound **8**. Figure S13. ^1^H-NMR spectrum of compound **9**. Figure S14. ^1^H-NMR spectrum of compound **10**. Figure S15. ^1^H-NMR spectrum of compound **11**. Figure S16. ^1^H-NMR spectrum of compound **12**. Figure S17. ^1^H-NMR spectrum of compound **13**. Figure S18. ^1^H-NMR spectrum of compound **14**. Figure S19. ^1^H-NMR spectrum of compound **15**. Figure S20. ^1^H-NMR spectrum of compound **16**. Figure S21. ^1^H-NMR spectrum of compound **17**. Figure S22. ^1^H-NMR spectrum of compound **18**. Figure S23. ^1^H-NMR spectrum of compound **19**. Figure S24. ^1^H-NMR spectrum of compound **20**. Figure S25. ^1^H-NMR spectrum of compound **21**. Figure S26. ^1^H-NMR spectrum of compound **22**. Figure S27. ^1^H-NMR spectrum of compound **23**. Figure S28. ^1^H-NMR spectrum of compound **24**. Figure S29. ^1^H-NMR spectrum of compound **25**. Figure S30. ^1^H-NMR spectrum of compound **26**. Figure S31. Structures of compounds **1**–**26**.

## Abbreviations

HPLC	High-performance liquid chromatography
HMBC	Heteronuclear multiple bond correlation
SAR	Structure–activity relationship
MTT	3-(4,5-dimethylthiazol-2-yl)-2,5-diphenyl tetrazolium bromide
FCM	Flow cytometry
Bcl-2	B-cell lymphoma 2
MMP	Matrix metalloproteinase
OD	Optical density
FITC	Fluorescein isothiocyanate
PI	Propidium iodide
DMSO	Dimethyl sulfoxide
