# Supplementary material for: Xanthones from the Bark of Garcinia xanthochymus and the Mechanism of Induced Apoptosis in Human Hepatocellular Carcinoma HepG2 Cells via the Mitochondrial Pathway
Source: Int J Mol Sci. 2019 Sep 27;20(19):4803. doi: 10.3390/ijms20194803 (PMC6801373; doi:10.3390/ijms20194803)
Supplement: Supplementary file 1 [file ijms-20-04803-s001.pdf]

Xanthones from the Bark of *Garcinia xanthochymus* and the Mechanism of Induced Apoptosis in Human Hepatocellular Carcinoma HepG2 Cells via the Mitochondrial Pathway

Shan Jin [1], Kuan Shi [1], Liu Liu [2], Yu Chen [2] and Guangzhong Yang [1,*]

[1] School of Pharmaceutical Sciences, South-Central University for Nationalities, Wuhan 430074, China
[2] College of Chemistry and Material Sciences, South-Central University for Nationalities, Wuhan 430074, China
* Correspondence: yangguangzhong@mail.scuec.edu.cn; Tel.: +86-27-67841196

Electronic Supplementary Information

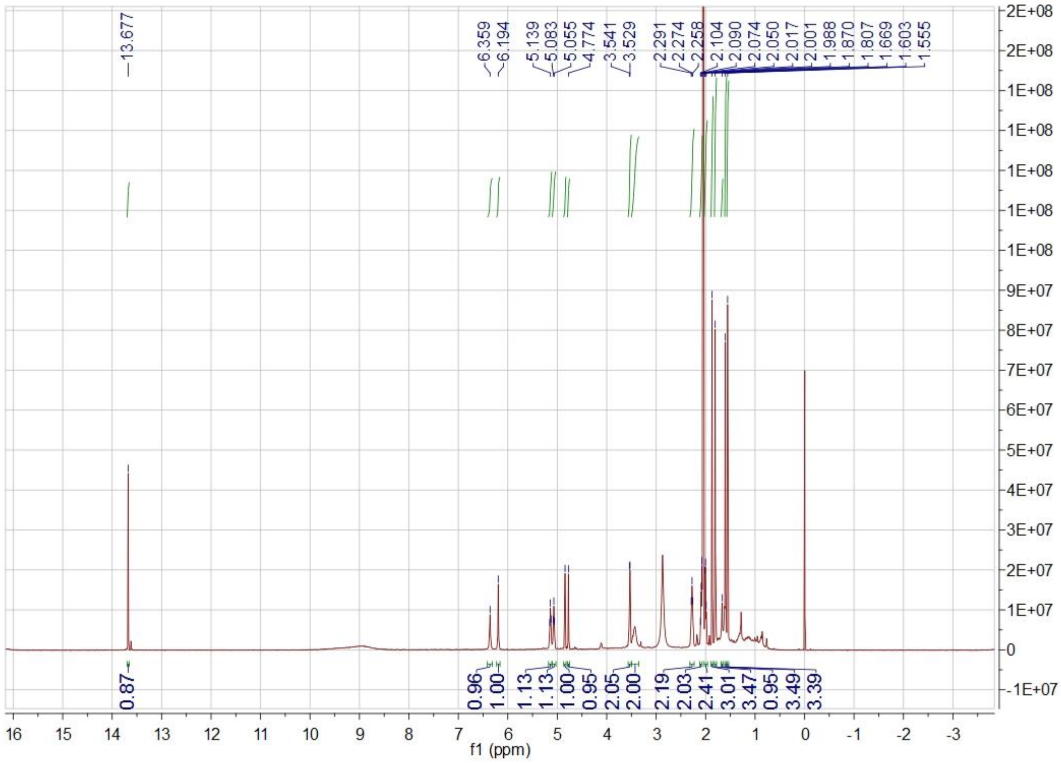

Figure S1. ^{1}H-NMR spectrum of compound **1.**

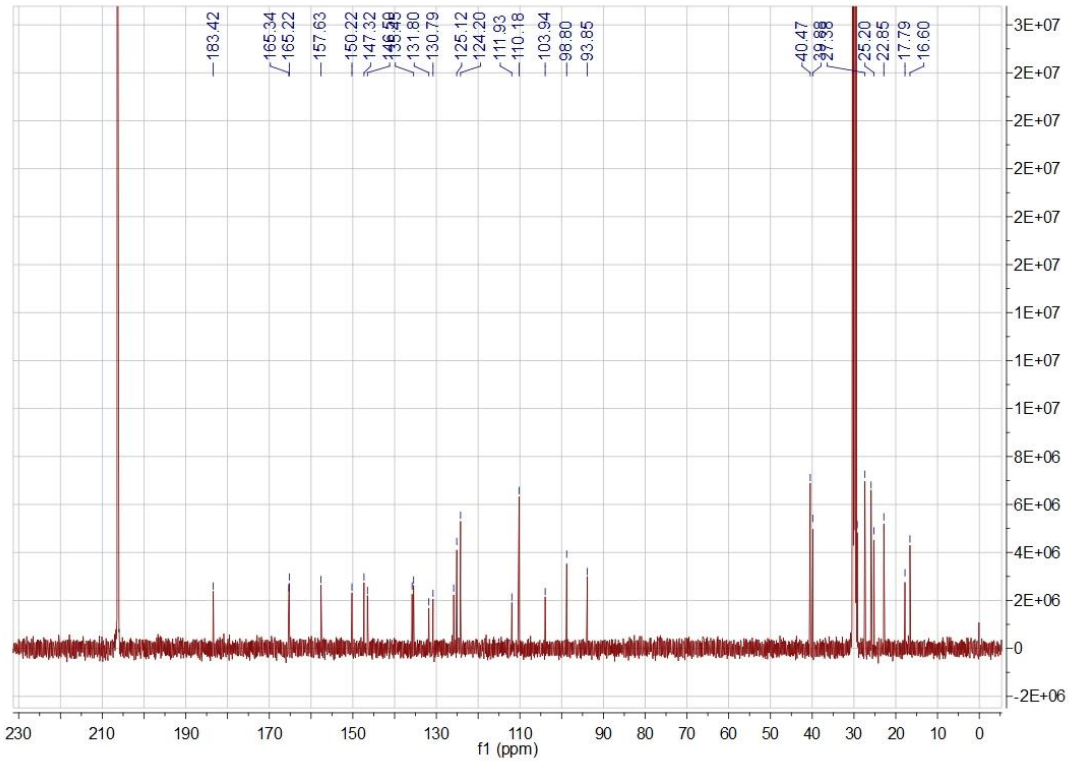

Figure S2. ^{13}C-NMR spectrum of compound **1.**

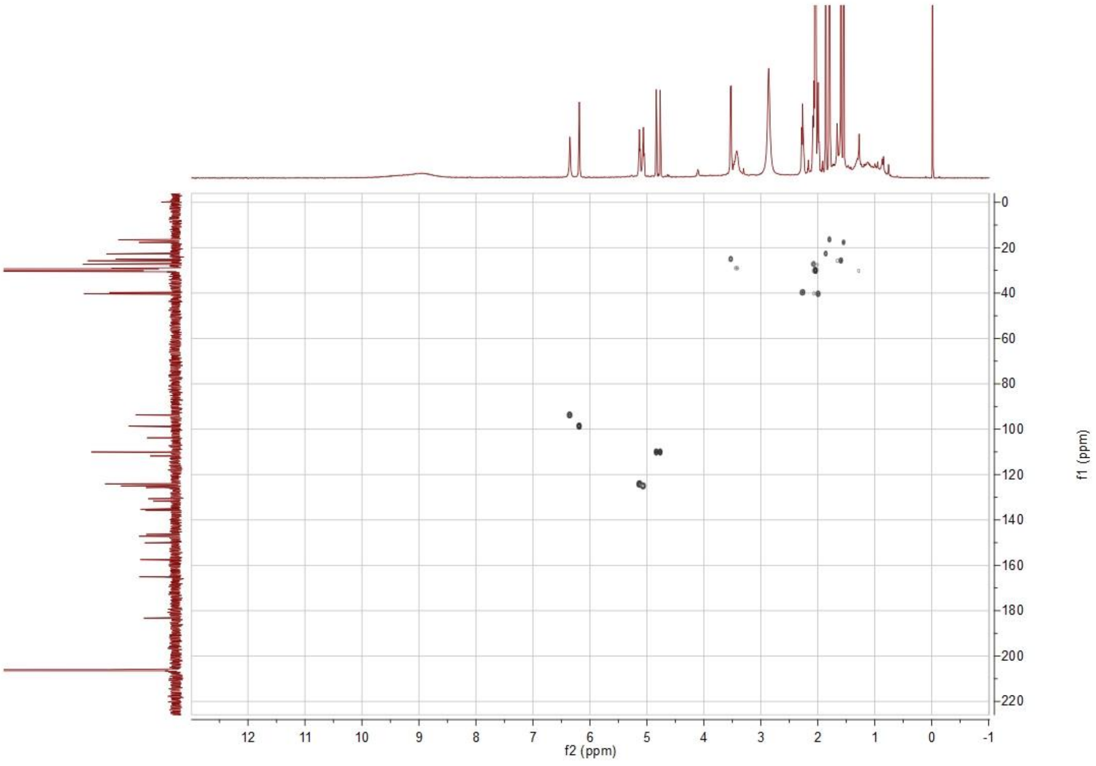

Figure S3. HSQC spectrum of compound **1.**

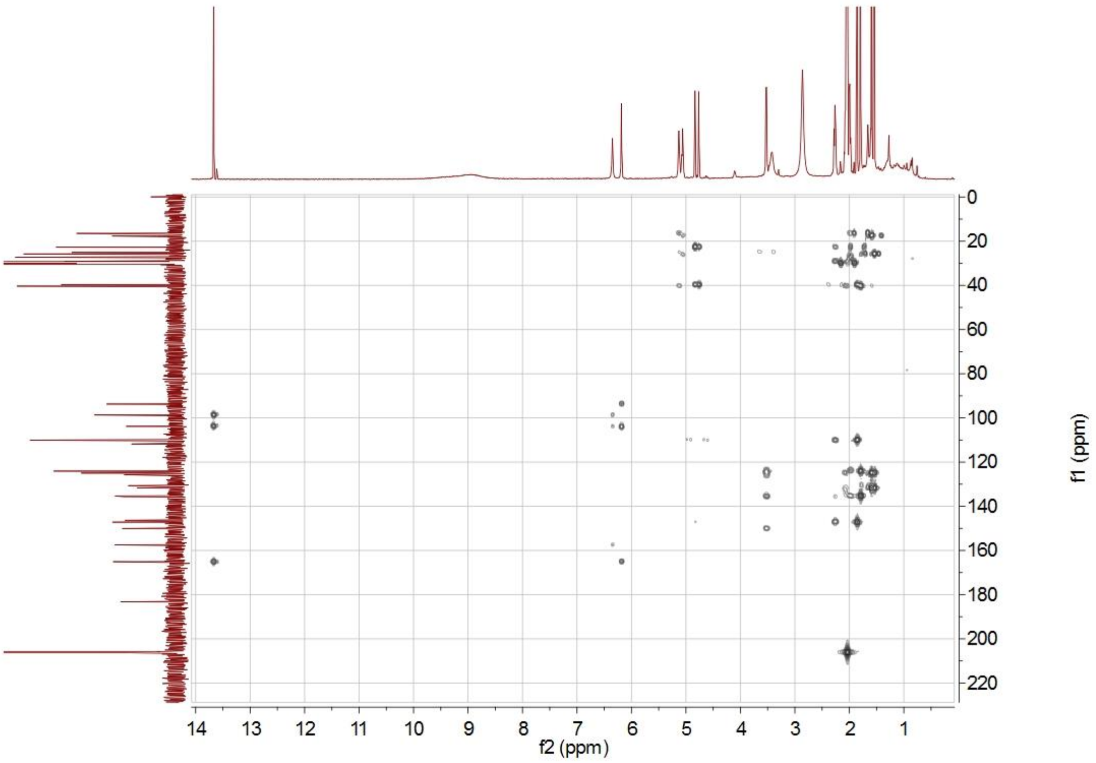

Figure S4. HMBC spectrum of compound **1.**

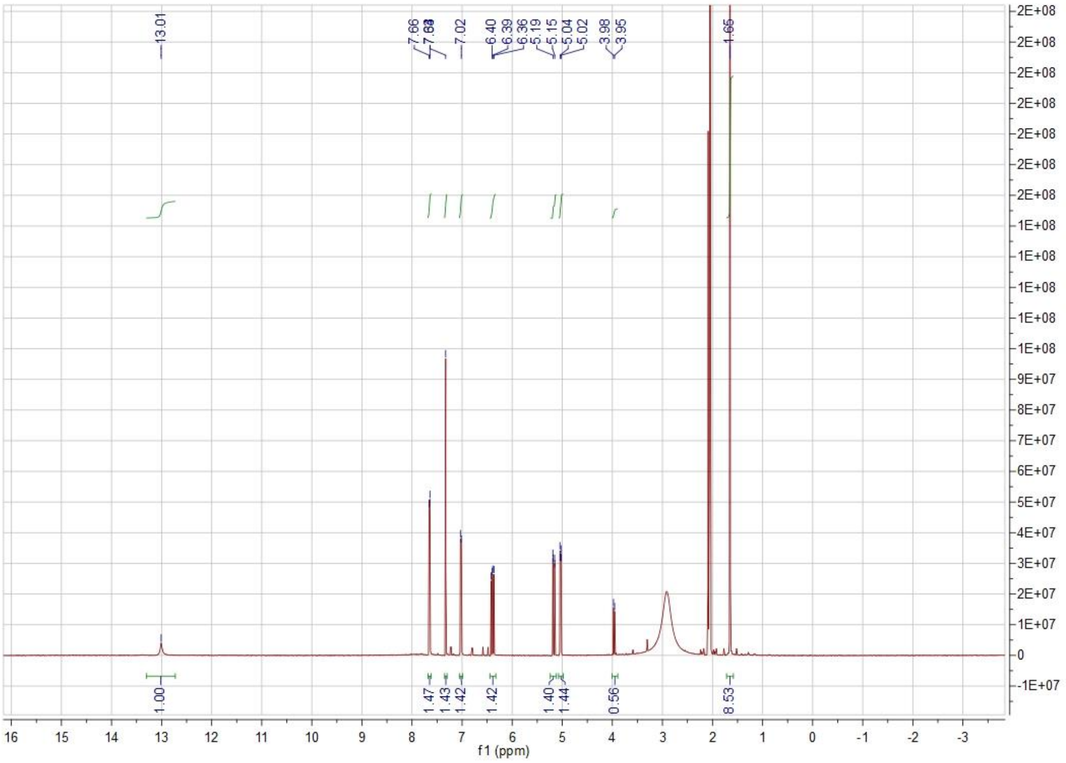

Figure S5. ^{1}H-NMR spectrum of compound **2.**

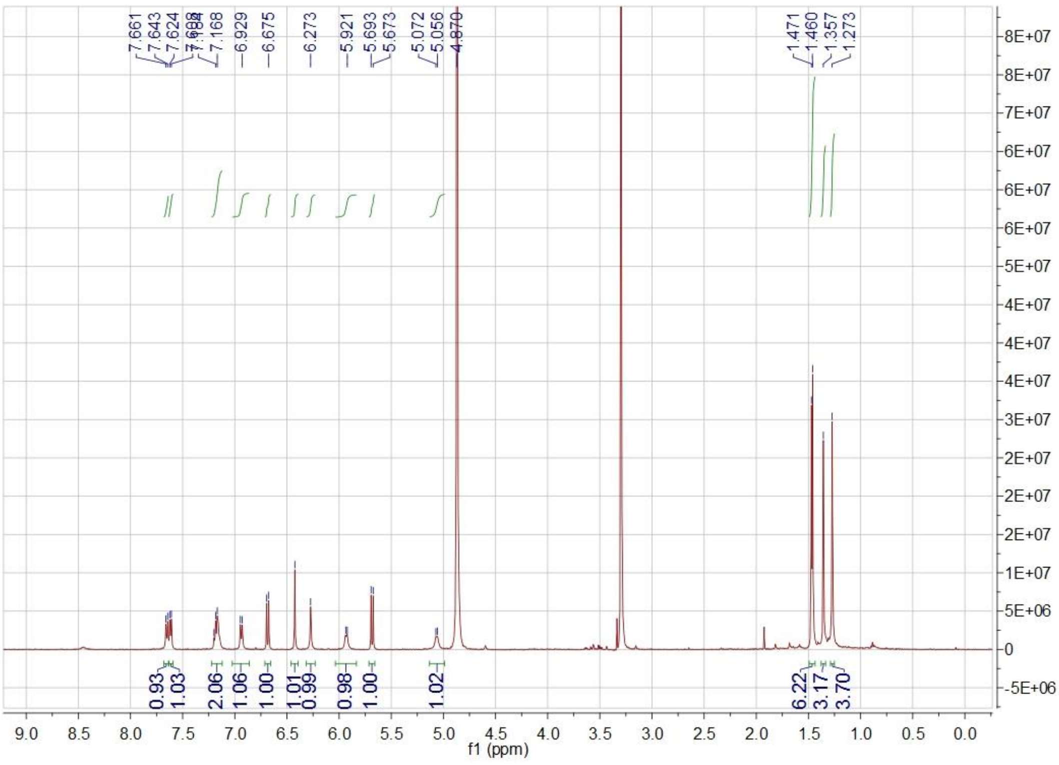

Figure S6. ^{1}H-NMR spectrum of compound **3.**

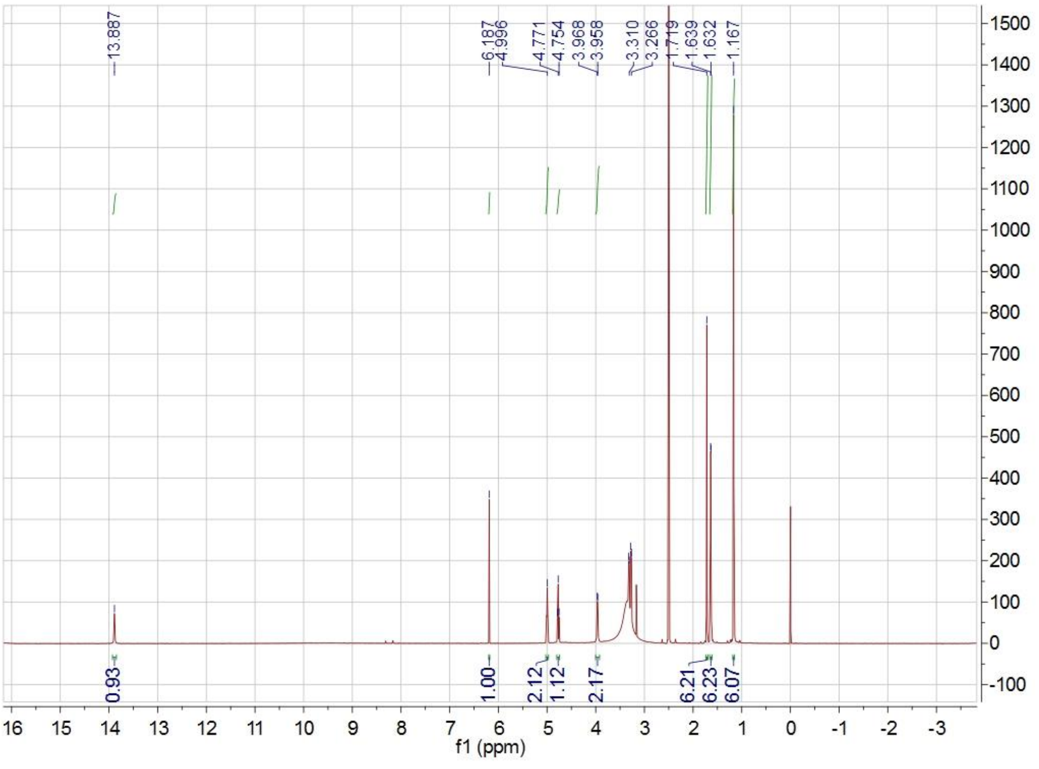

Figure S7. ^{1}H-NMR spectrum of compound **4.**

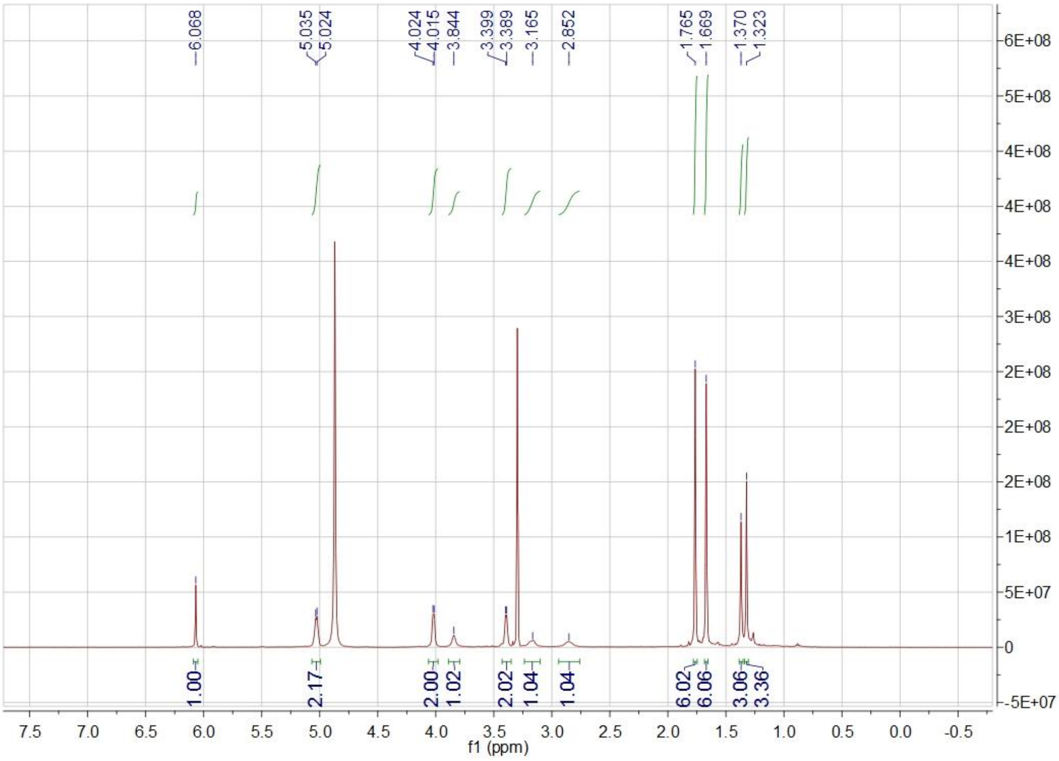

Figure S8. ^{1}H-NMR spectrum of compound **5.**

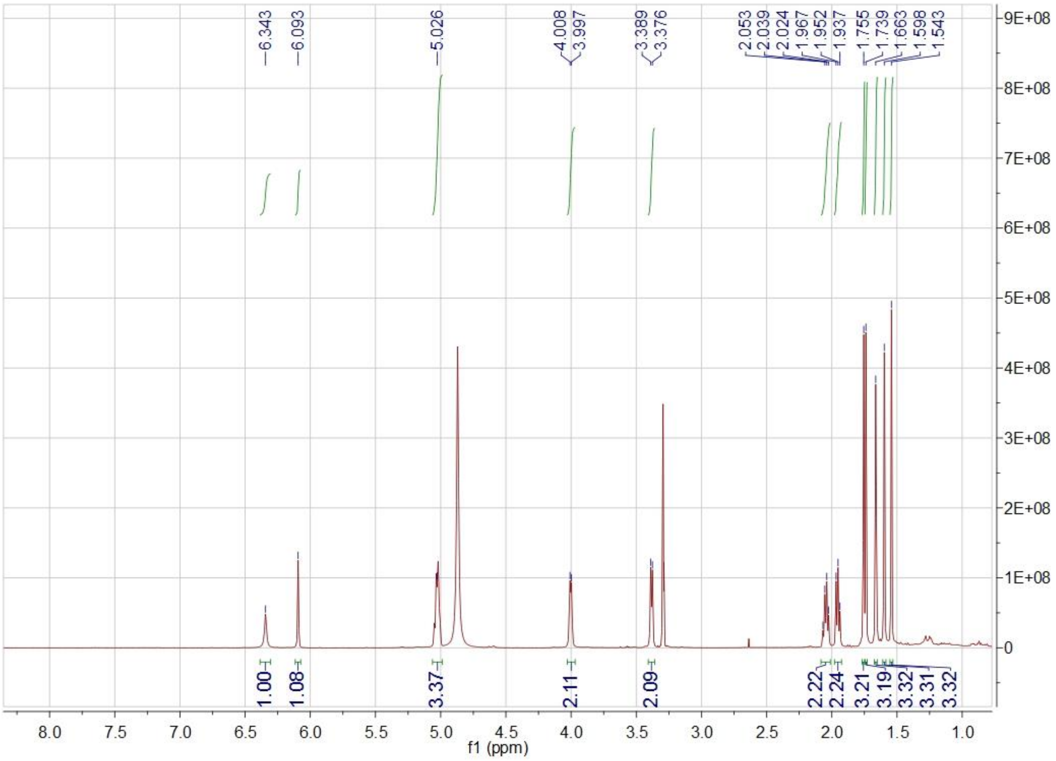

Figure S9. ^{1}H-NMR spectrum of compound **6.**

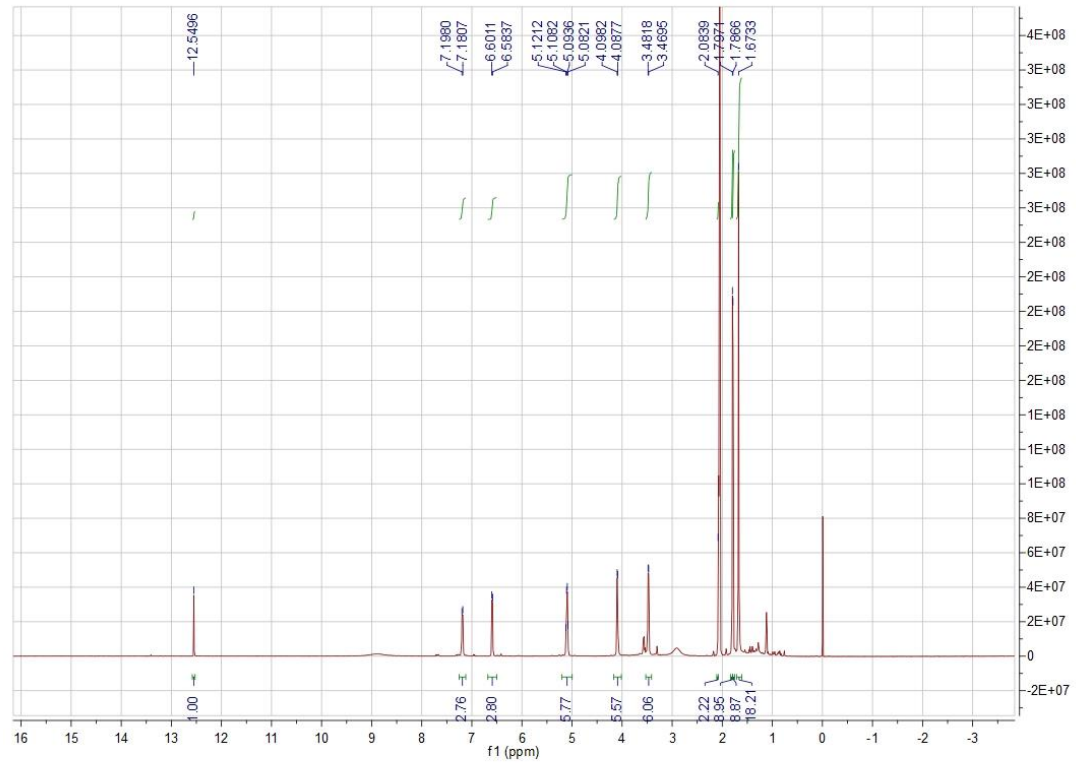

Figure S10. ^{1}H-NMR spectrum of compound **7.**

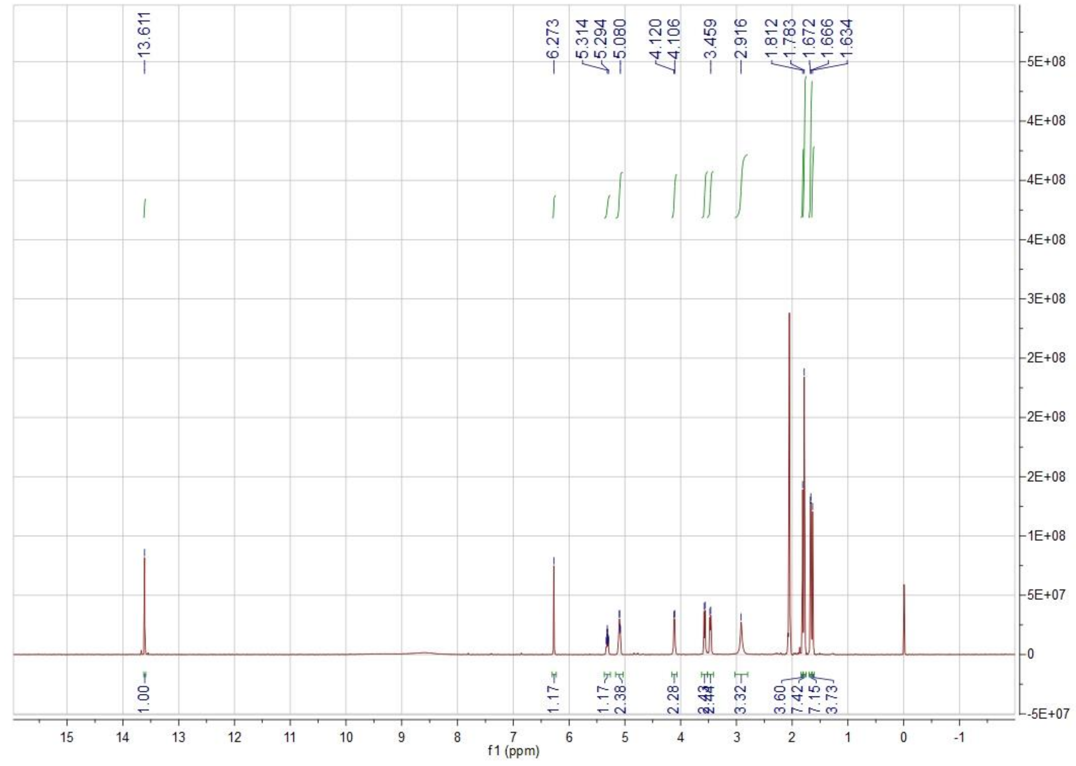

Figure S11. ^{1}H-NMR spectrum of compound **8.**

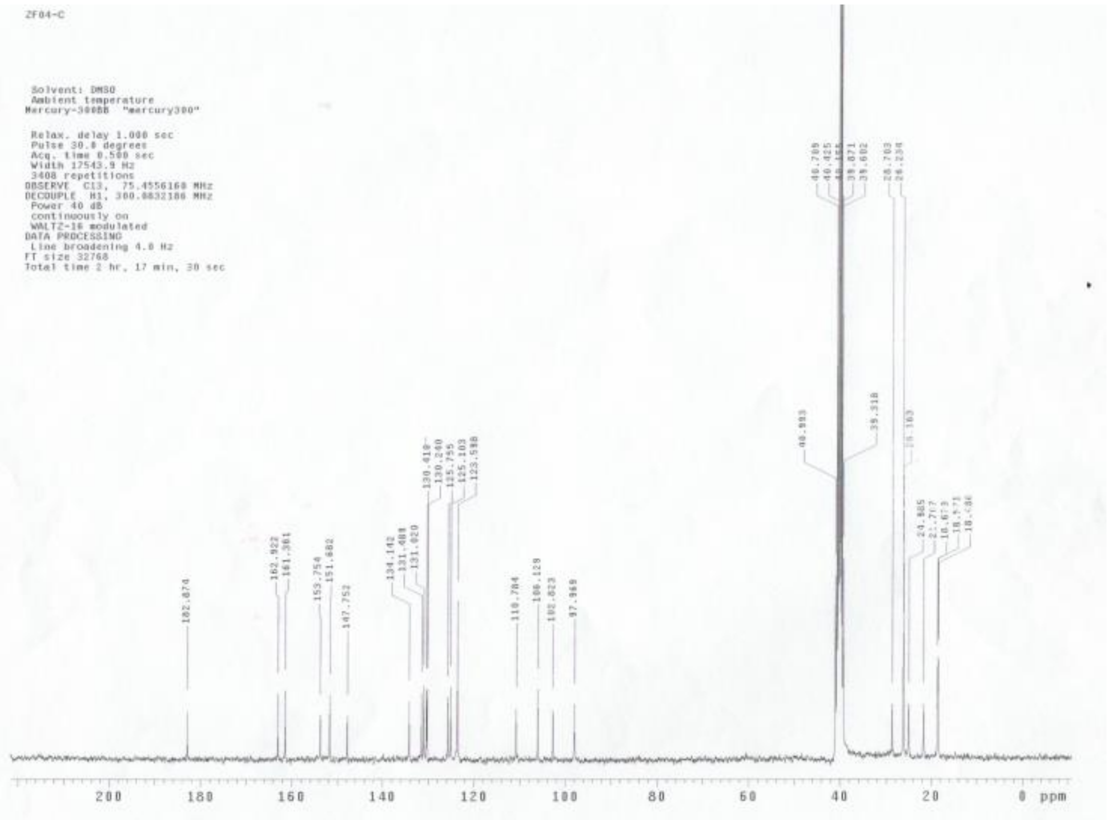

Figure S12. ^{13}C-NMR spectrum of compound **8.**

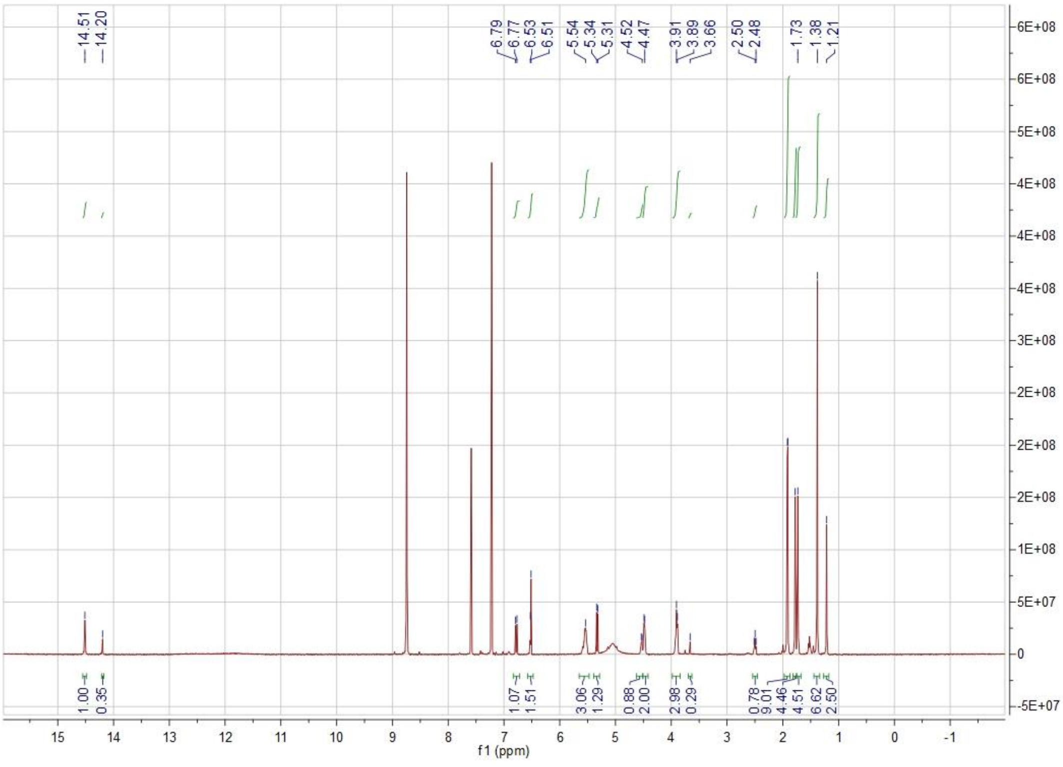

Figure S13. ^{1}H-NMR spectrum of compound **9.**

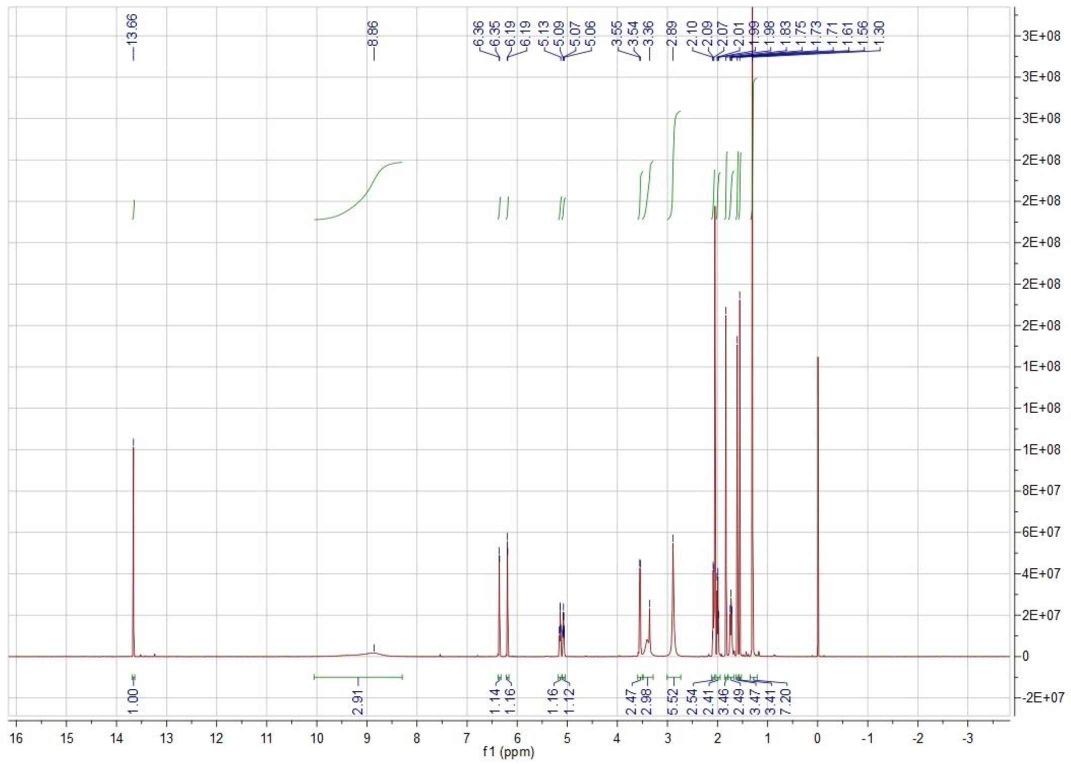

Figure S14. ^{1}H-NMR spectrum of compound **10.**

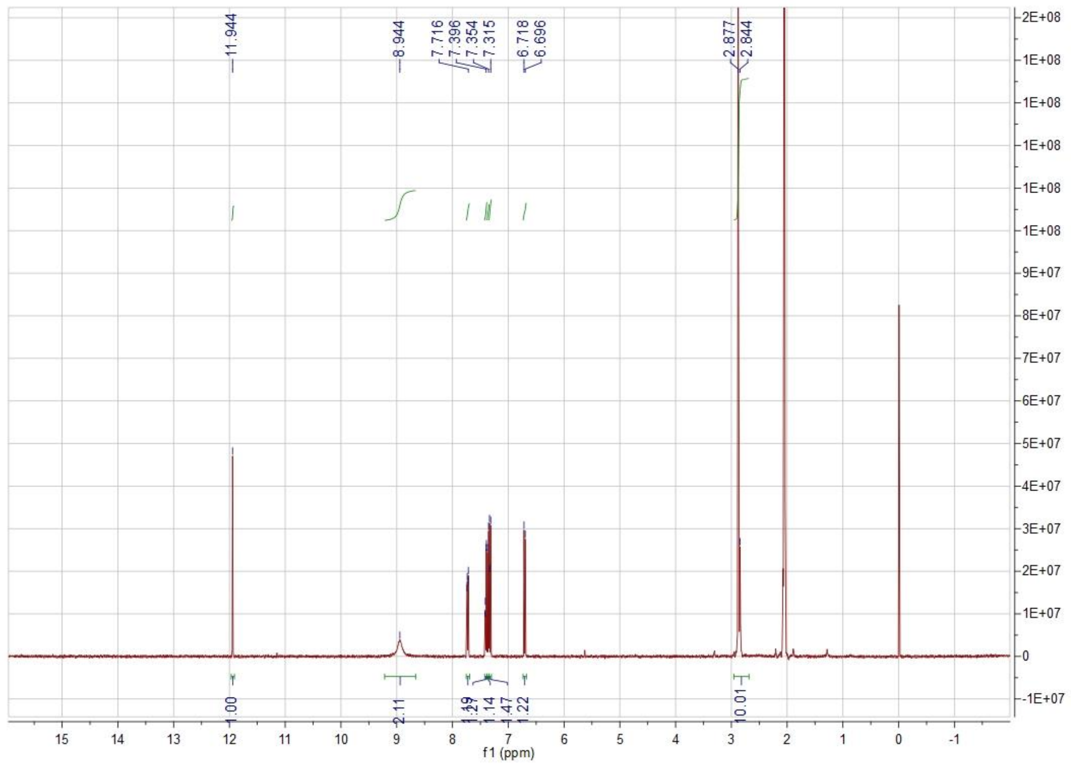

Figure S15. ^{1}H-NMR spectrum of compound **11.**

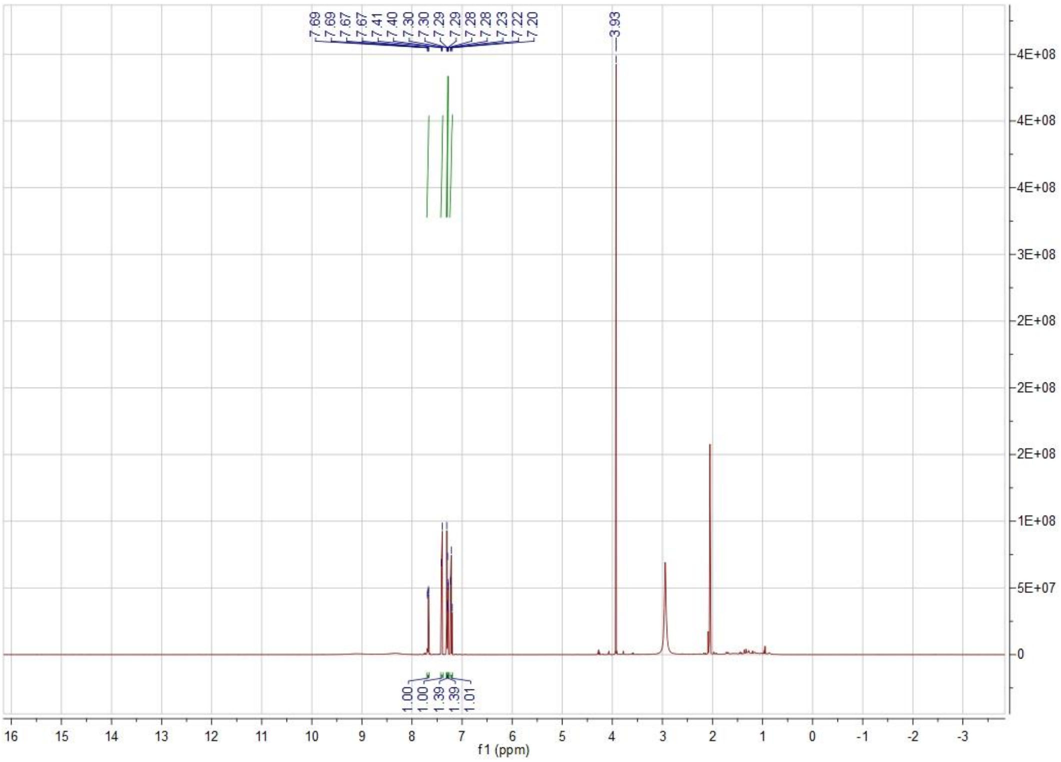

Figure S16. ^{1}H-NMR spectrum of compound **12.**

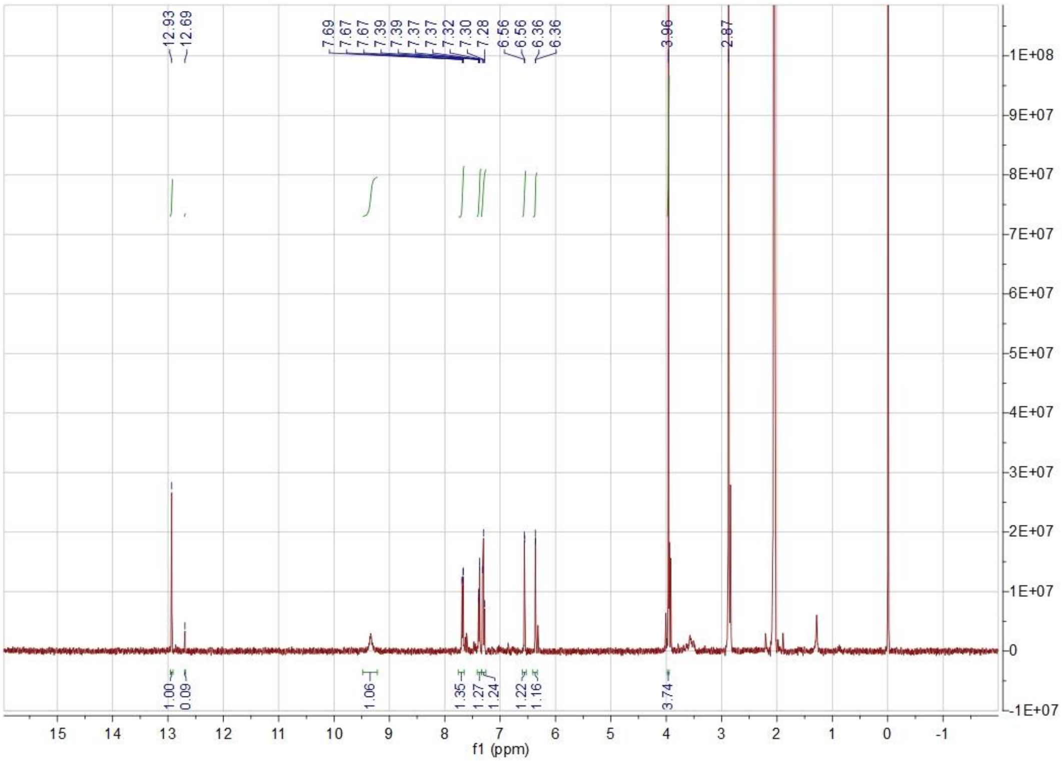

Figure S17. ^{1}H-NMR spectrum of compound **13.**

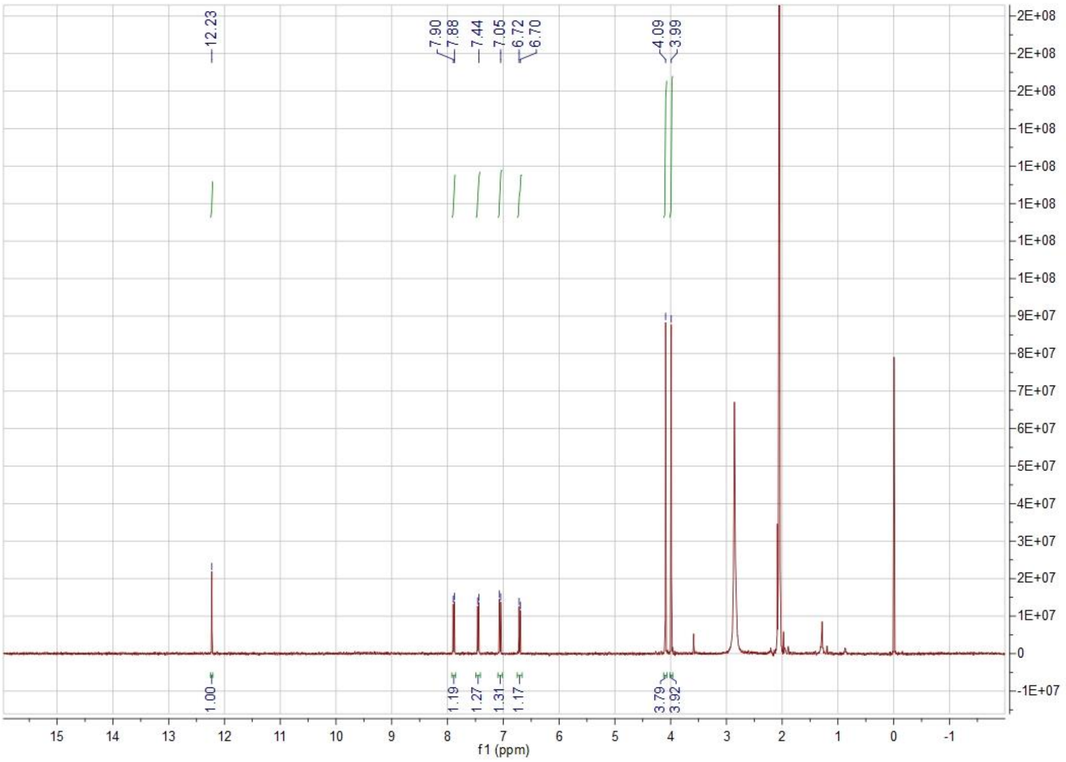

Figure S18. ^{1}H-NMR spectrum of compound **14.**

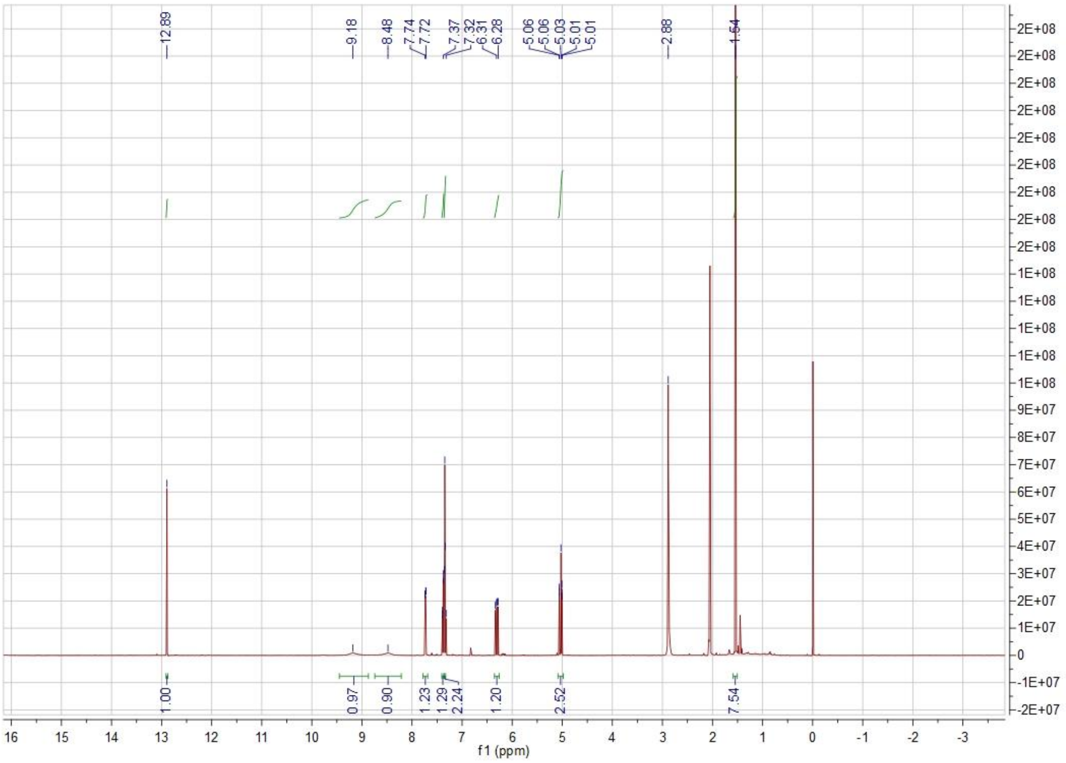

Figure S19. ^{1}H-NMR spectrum of compound **15.**

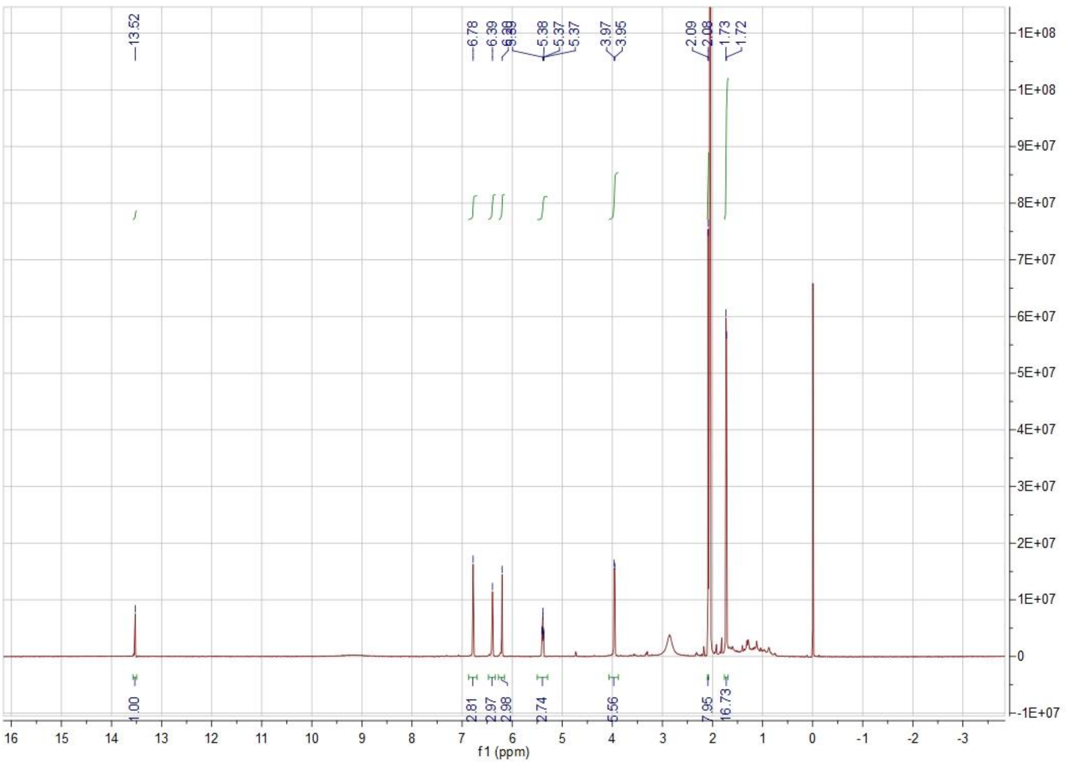

Figure S20. ^{1}H-NMR spectrum of compound **16.**

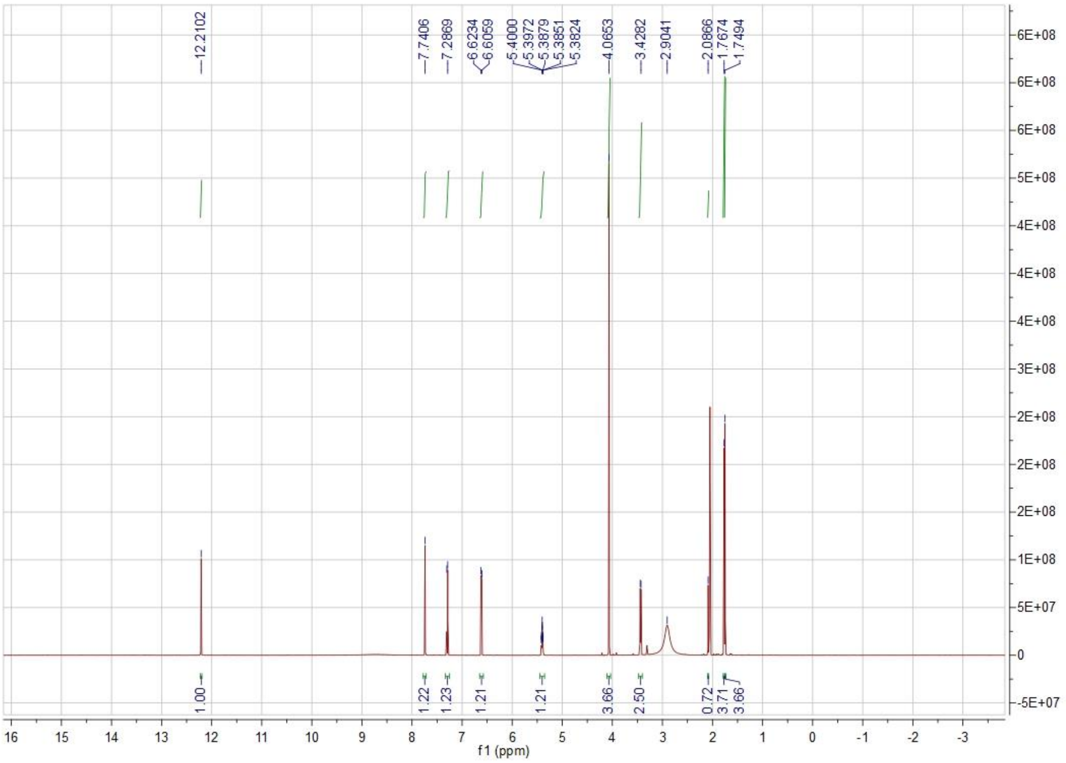

Figure S21. ^{1}H-NMR spectrum of compound **17.**

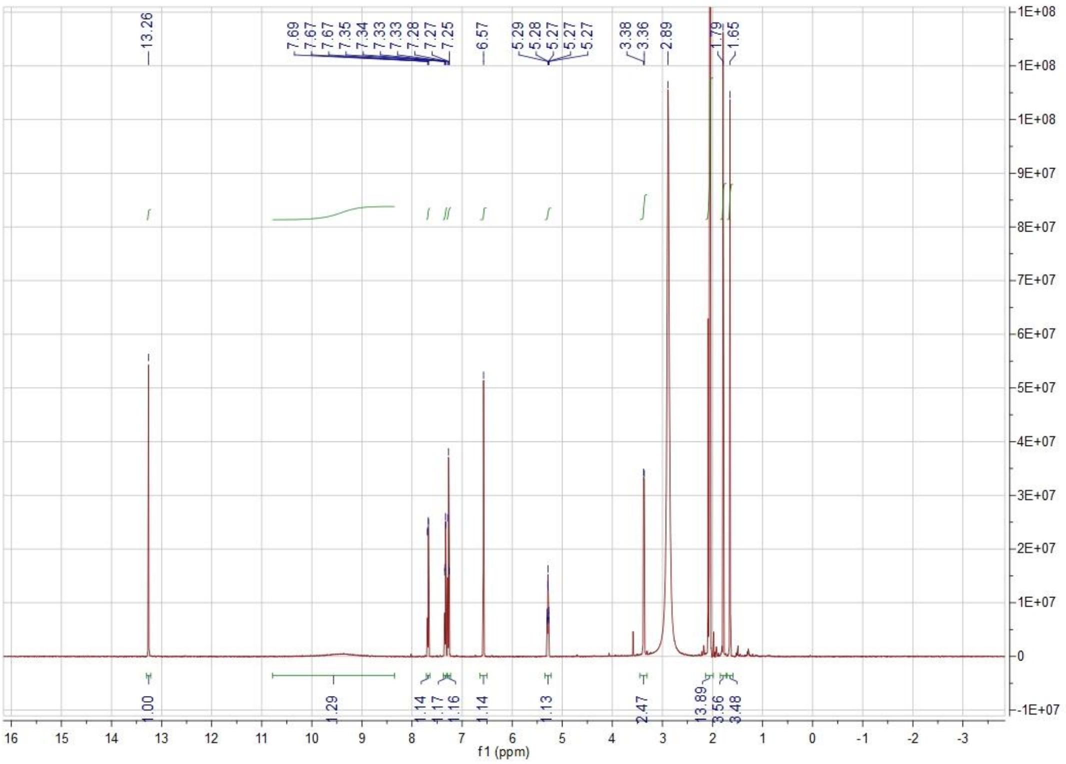

Figure S22. ^{1}H-NMR spectrum of compound **18.**

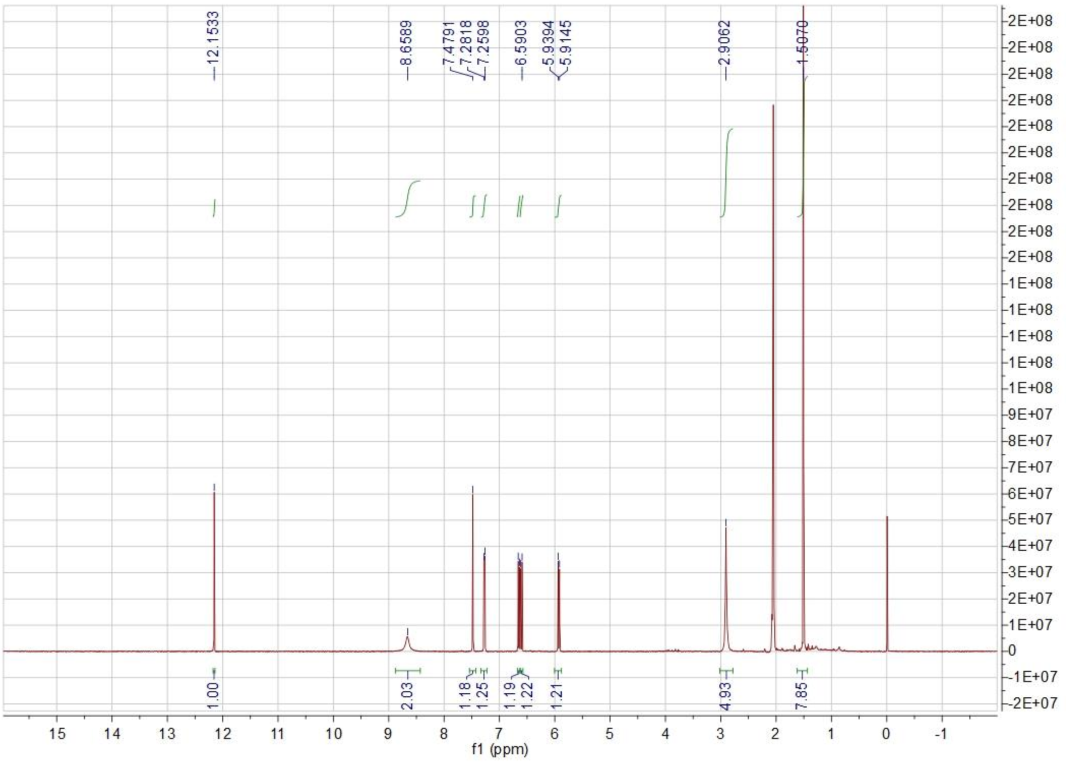

Figure S23. ^{1}H-NMR spectrum of compound **19.**

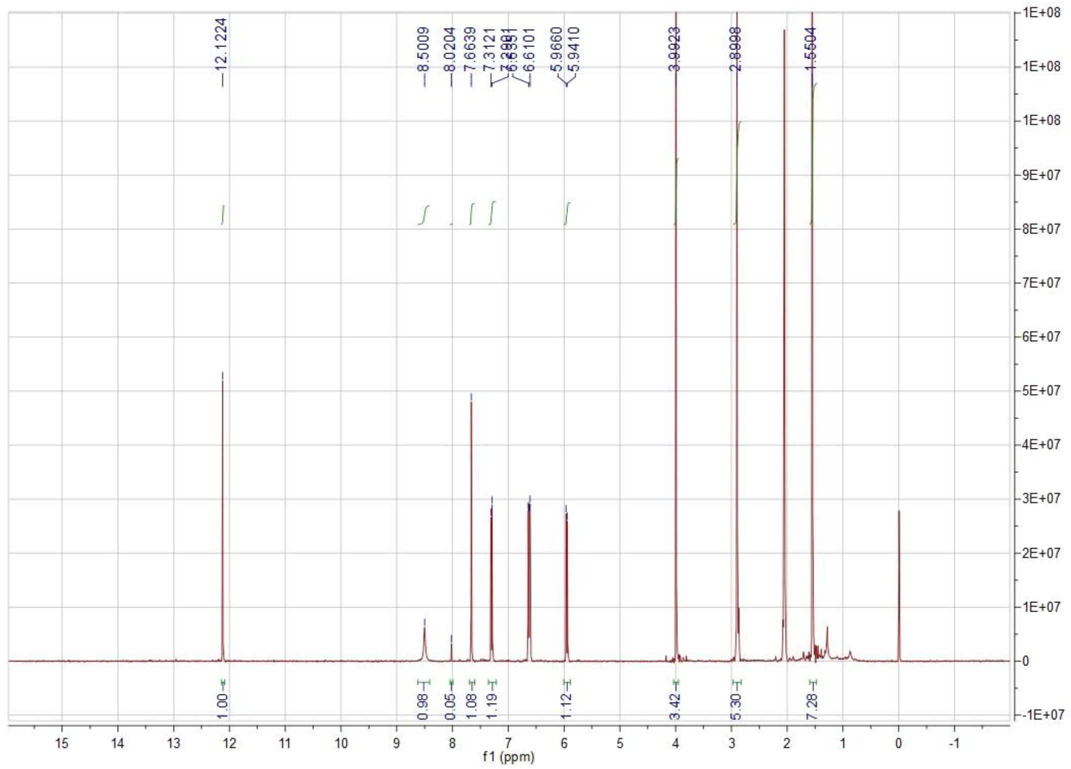

Figure S24. ^{1}H-NMR spectrum of compound **20.**

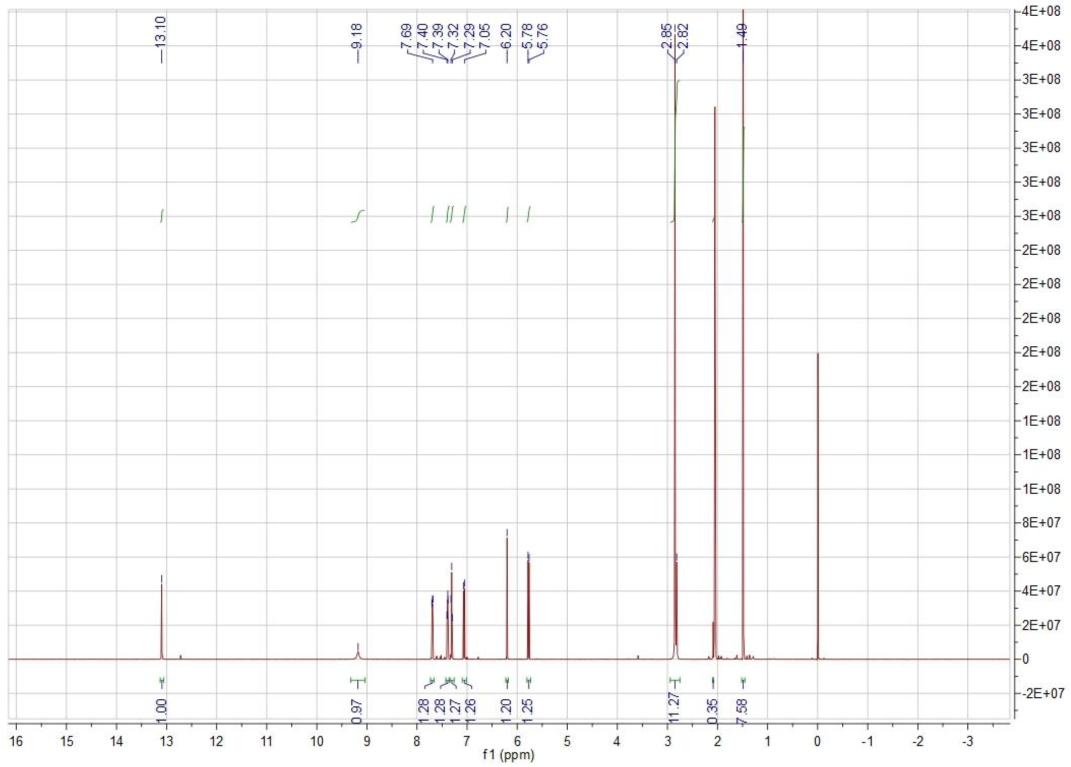

Figure S25. ^{1}H-NMR spectrum of compound **21.**

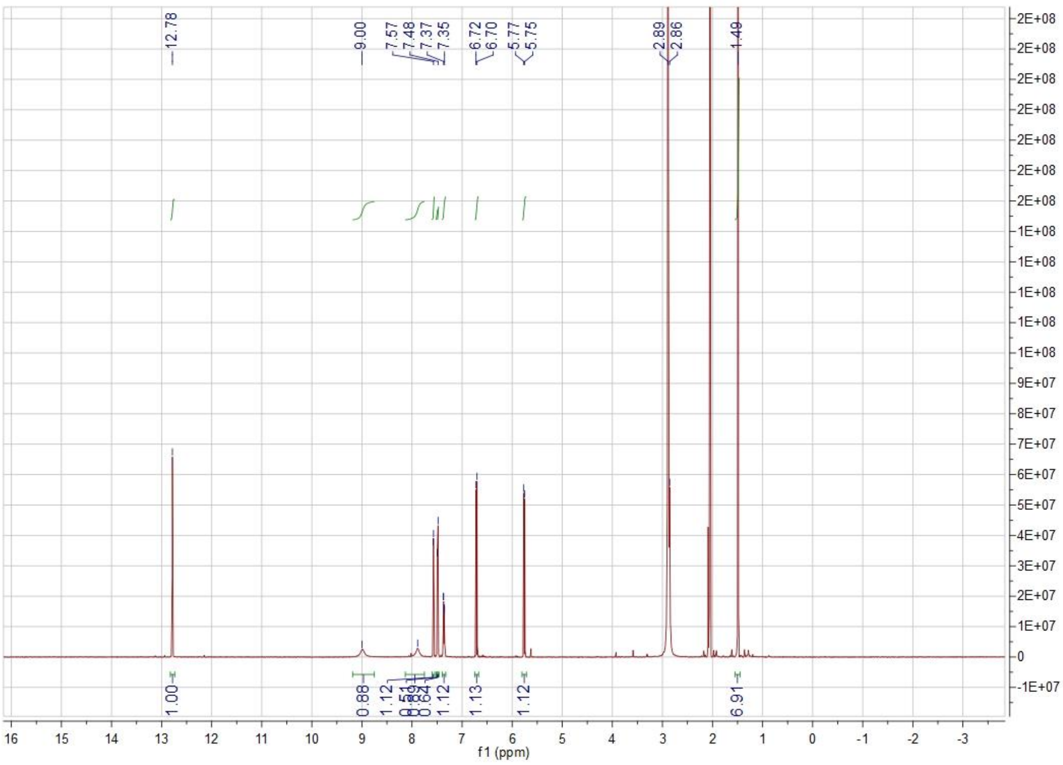

Figure S26. ^{1}H-NMR spectrum of compound **22.**

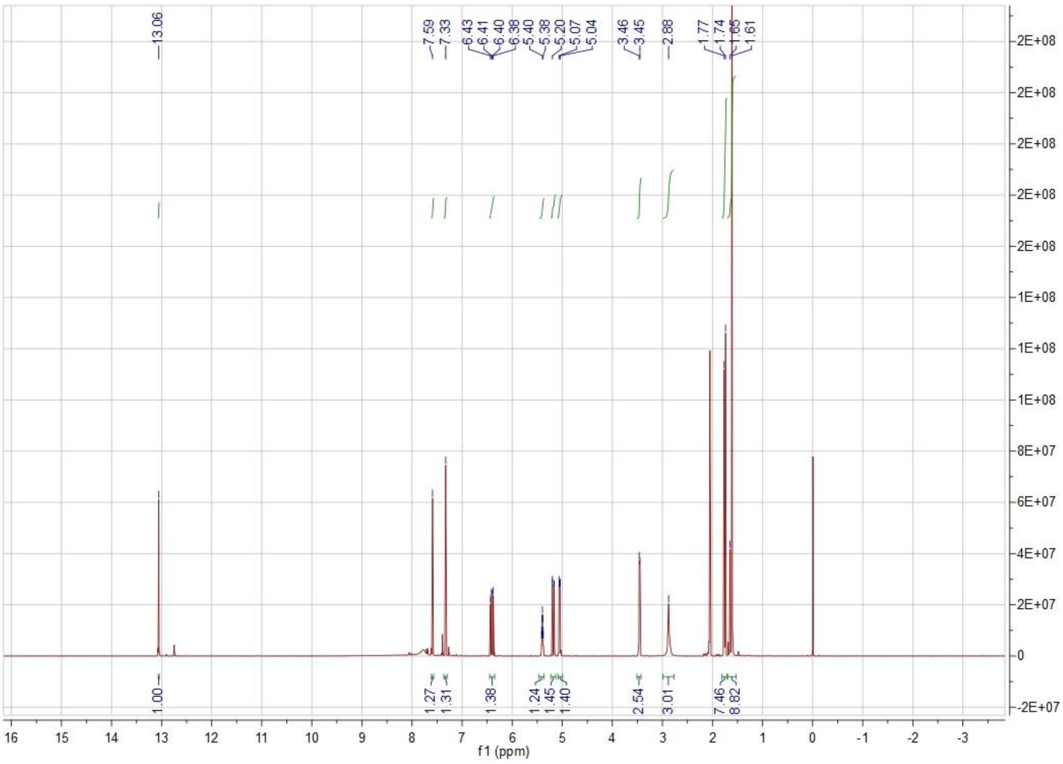

Figure S27. ^{1}H-NMR spectrum of compound **23.**

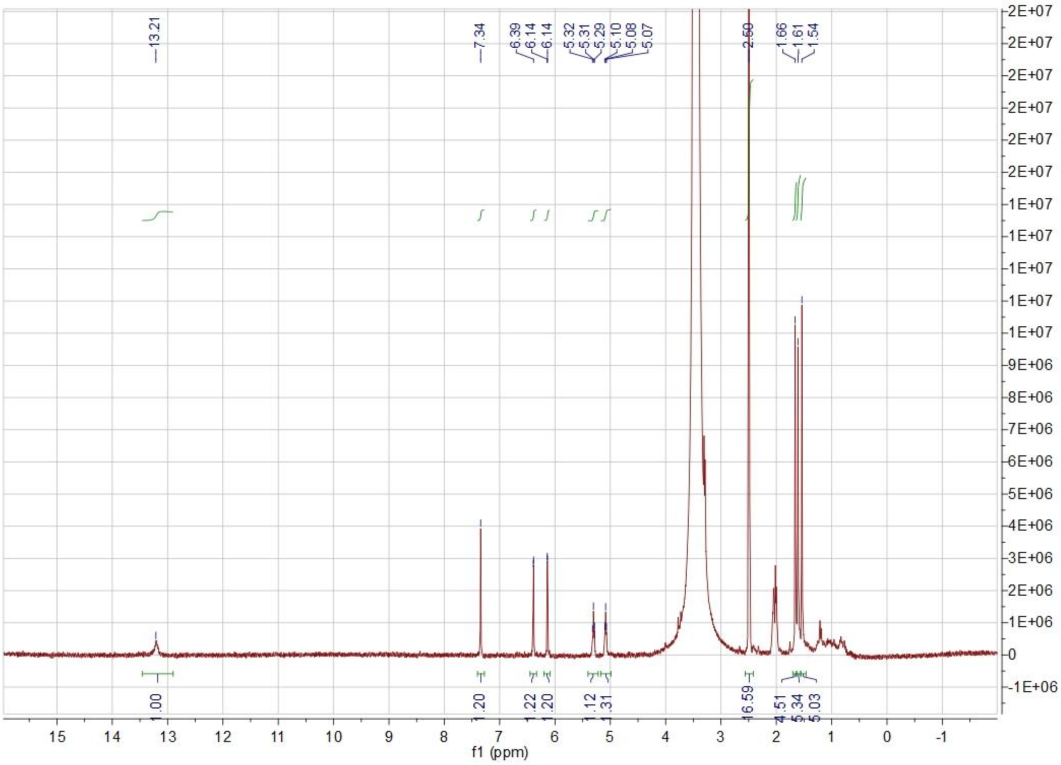

Figure S28. ^{1}H-NMR spectrum of compound **24.**

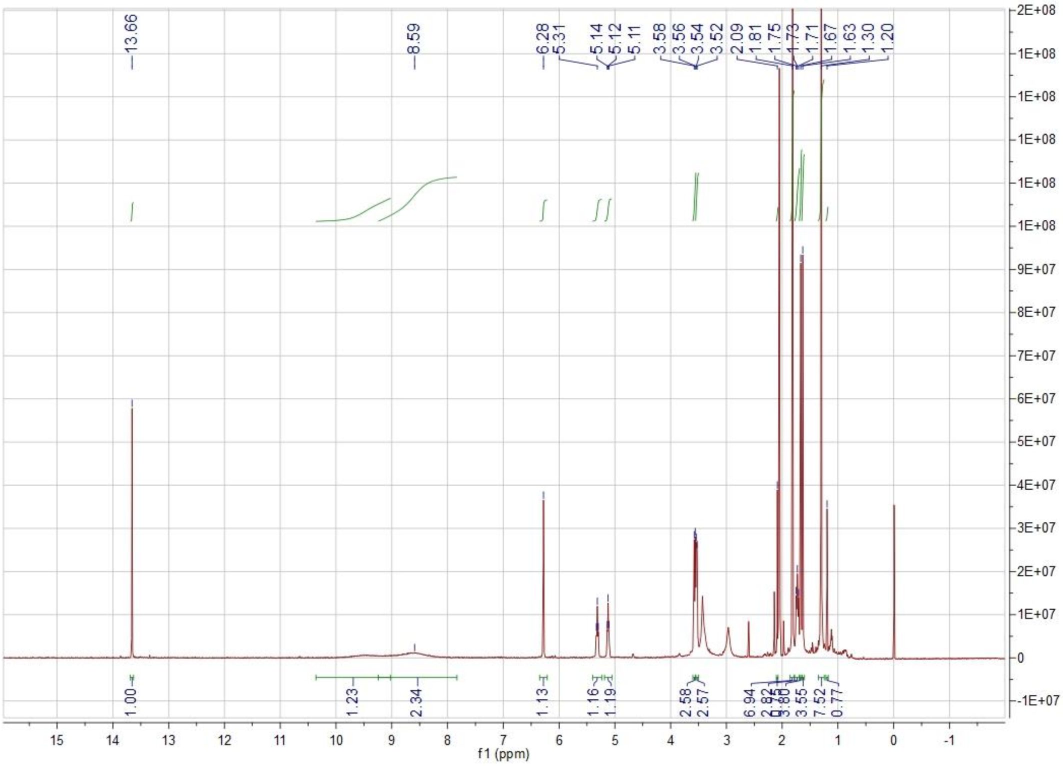

Figure S29. ^{1}H-NMR spectrum of compound **25.**

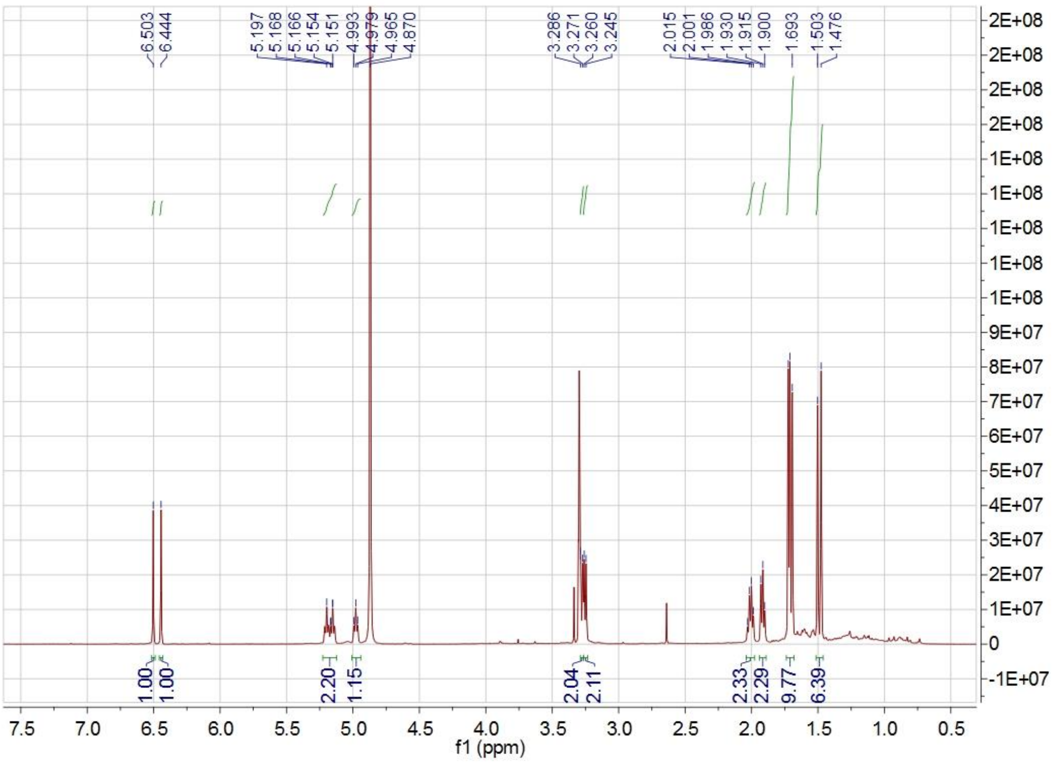

Figure S30. ^{1}H-NMR spectrum of compound **26.**

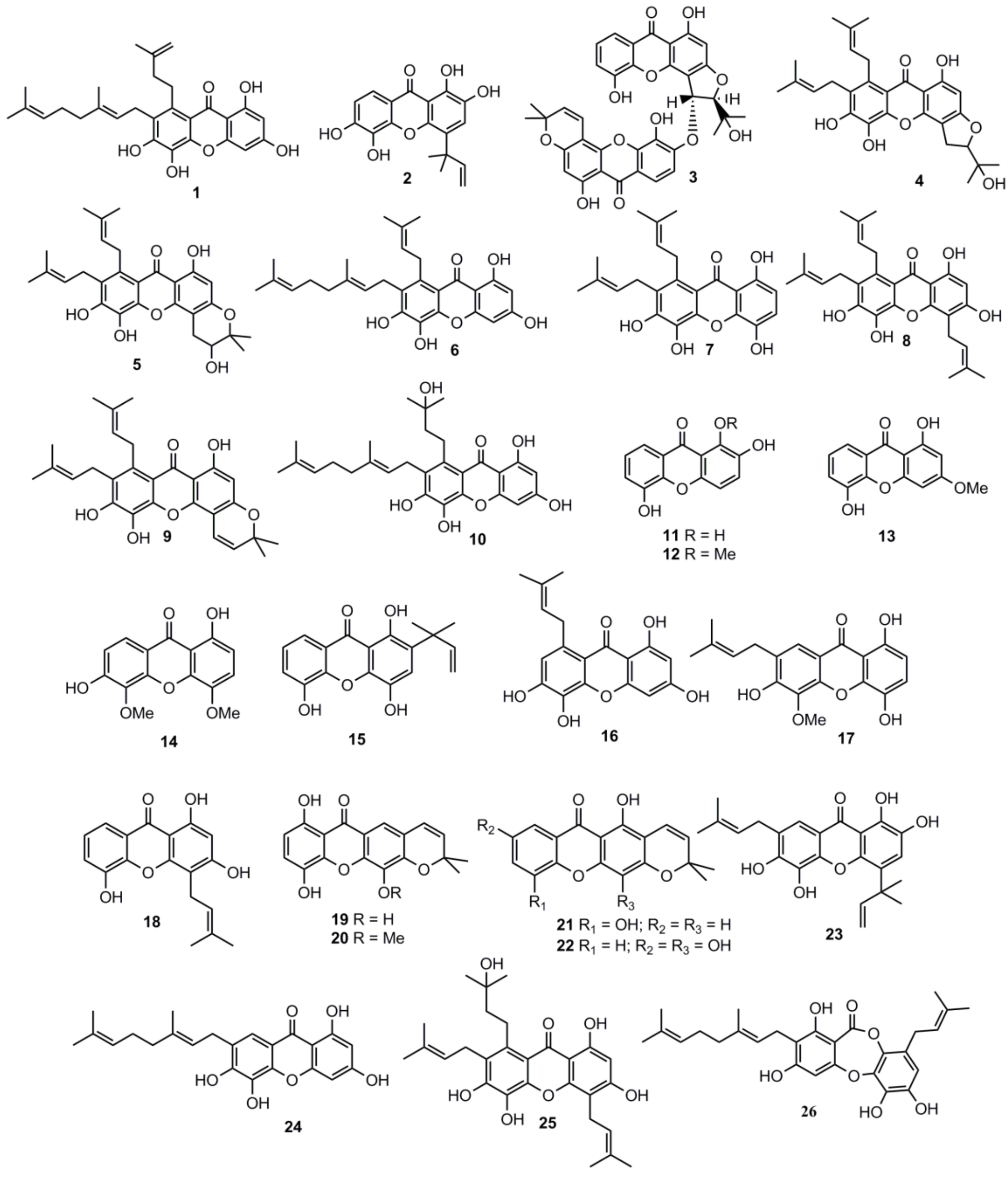

Figure S31. Structures of compounds **1**-**26.**
